# Electrocatalytic Depolymerization of Self-Immolative Poly(Dithiothreitol) Derivatives

**DOI:** 10.3390/molecules27196292

**Published:** 2022-09-23

**Authors:** Magnus Hansen-Felby, Steen U. Pedersen, Kim Daasbjerg

**Affiliations:** 1 Department of Chemistry and Interdiciplinary Nanoscience Center (iNANO), Aarhus University, Langelandsgade 140, DK-8000 Aarhus C, Denmark; 2 Novo Nordisk Foundation CO2 Research Center, Aarhus University, Gustav Wieds Vej 14, DK-8000 Aarhus C, Denmark

**Keywords:** self-immolative, electrocatalytic, depolymerization, polymer, poly(dithiothreitol), anthraquinone, disulfide, radical anion

## Abstract

We report the use of electrogenerated anthraquinone radical anion (AQ•^−^) to trigger fast catalytic depolymerization of polymers derived from poly(dithiothreitol) (pDTT)—a self-immolative polymer (SIP) with a backbone of dithiothreitols connected with disulfide bonds and end-capped via disulfide bonds to pyridyl groups. The pDTT derivatives studied include polymers with simple thiohexyl end-caps or modified with AQ or methyl groups by Steglich esterification. All polymers were shown to be depolymerized using catalytic amounts of electrons delivered by AQ•^−^. For pDTT, as little as 0.2 electrons per polymer chain was needed to achieve complete depolymerization. We hypothesize that the reaction proceeds with AQ•^−^ as an electron carrier (either molecularly or as a pendant group), which transfers an electron to a disulfide bond in the polymer in a dissociative manner, generating a thiyl radical and a thiolate. The rapid and catalytic depolymerization is driven by thiyl radicals attacking other disulfide bonds internally or between pDTT chains in a chain reaction. Electrochemical triggering works as a general method for initiating depolymerization of pDTT derivatives and may likely also be used for depolymerization of other disulfide polymers.

## 1. Introduction

Self-immolative polymers (SIPs) are a specific type of degradable polymer. These polymers undergo end-to-end depolymerization in response to an external stimulus [1]. In contrast to conventional degradable polymers, such as polyesters, SIPs only need one trigger event/reagent per polymer chain to impose full depolymerization. Upon cleavage of a stimuli-responsive end-cap, a reactive terminus is exposed, which then undergoes depolymerization into specific small molecules through a cascade reaction [2]. The depolymerization products can, for some polymers, be recovered as small monomer-like compounds from which the monomer can be regenerated and used for the synthesis of pristine polymer materials [3]. A comprehensive review was published by Gillies and coworkers in 2019 discussing applications and various triggering stimuli [2], e.g., UV–Vis [4], fluoride [5,6,7,8], heat [9], bovine serum albumin [10], acid [11,12], palladium [6,7,8], base [7], and H_2_O_2_ [13], among others. Phillips and coworkers developed a system comprising poly(benzyl carbamate), which depolymerized in response to metal ions, i.e., Hg^2+^ and Pb^2+^ [14]. The polymer was equipped with an H_2_O_2_-sensitive aryl boronate end-cap, and bead-bound proteins generated H_2_O_2_ in the presence of glucose and metal ions, triggering depolymerization.

White and coworkers encapsulated electronic circuits in a blend of cyclic poly(phthalaldehyde) and polytetrafluoroethylene together with a molecular photoacid generator [11]. Upon exposure to UV light, the photoacid generated hydrochloric acid, which triggered depolymerization of the poly(phthalaldehyde). More recently, Moore and coworkers have shown that SIP depolymerization of cyclic poly(phthalaldehyde) could be triggered using an intermolecular photo redox catalyst to mediate electron transfer [15]. This allowed for depolymerization of solid-state materials.

Depolymerization of SIPs can also be triggered for certain polymers through cleavage of specific side groups. Phillips and coworkers showed that poly(benzyl ethers) with silyl, or allyl protecting groups attached on the sidechains, could depolymerize through cleavage of the sidechain protecting groups [16]. The extent of depolymerization was dependent on the specific site at which cleavage occurred as the degradation only propagated down-chain from the cleavage site. By increasing the concentration of cleavage sites, these modifications allowed for a much higher depolymerization rate, enabling depolymerization of solid-state materials in a few minutes. Zhang and coworkers considered a similar approach using poly(benzyl ethers) equipped with cyclization spacers on the side groups. They post-functionalized the polymers with polyethyleneglycol with one or two thiol groups, which enabled synthesis of crosslinked materials, which, in response to reductive conditions, could depolymerize fully [17]. Recently, Gillies and coworkers reported a diblock of poly(ethyl glyoxylate) and different polyglyoxylamides, with each block having a distinct triggering stimulus. This made possible the selective end-cap cleavage and subsequent depolymerization of one block while leaving the other block intact [18].

Application of electrochemistry to depolymerize plastics is limited to a few examples, including lignin [19,20,21,22,23] and 2,5-dimercapto-1,3,4-thiadiazole polymers [24,25]. Some conventional plastics, such as polypropylene, have been converted into fuels by combining electrochemistry with pyrolysis [26]. Cellulose can undergo electrocatalytic degradation in 0.5 M sulfuric acid solution, although the extent of depolymerization is limited and with poor product selectivity [27]. Poly(ethylene terephthalate) can be converted into its monomers, ethylene glycol and terephthalic acid, by electrochemically generated hydroxide [28].

Various triggering approaches have been used in the SIP field, but the use of electrochemistry to induce depolymerization is lacking. Recently, we published that poly(dithiothreitol) (pDTT) with pendant anthraquinone (AQ) groups exhibited complete depolymerization in response to electrochemical reduction of AQ to AQ•^−^ in what was the first use of electrochemically induced depolymerization triggering of SIPs [29]. Alternatively, dithiothreitol and base (triethylamine) can be used to initiate depolymerization [29,30,31,32]. An interesting aspect of the indirect electron transfer (or redox catalysis) [33,34,35] approach for carrying out reduction processes involving bond fragmentation is that it can lower significantly the electrochemical potential needed for accomplishing the process, and in a very controlled fashion. This was already shown in thorough investigations on both direct and indirect reductions of simple diaryl and dialkyl disulfides [36,37,38,39].

**Scheme 1:**
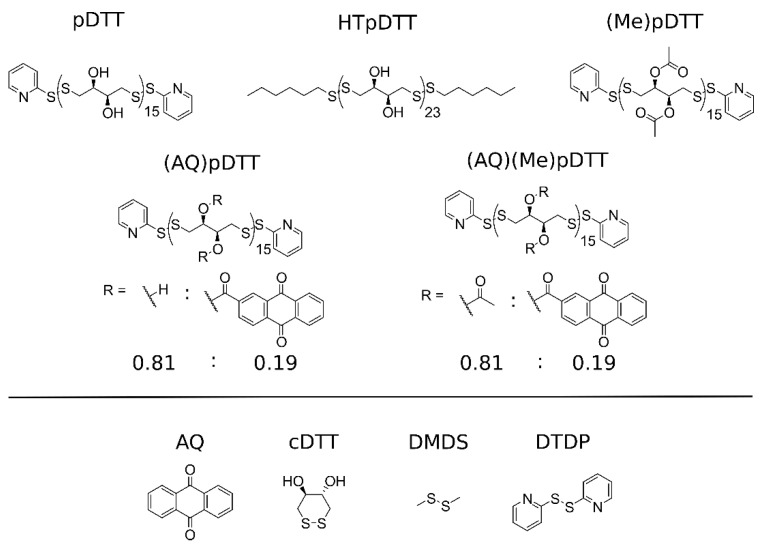
Chart of poly(dithiothreitol) derivatives (**top**) and model compounds (**bottom**).

In this work, we expand the applicability of this discovery and test electrotriggered depolymerization on a selection of four derivatized pDTTs together with pDTT itself (see top of Scheme 1). The investigation is conducted using the mediating effect of AQ in solution or as pendant AQ groups in the polymer, i.e., (AQ)pDTT or (AQ)(Me)pDTT. Steglich-modified pDTT with pendant acetyl groups, (Me)pDTT, was included in this study to mask the hydroxyl groups in order to reduce the risk of protonating AQ•^−^. As shown herein, electrochemical triggering leads to the catalytic depolymerization of all tested pDTT derivatives. We envision that these discoveries will open new avenues for rapid drug delivery and strong signal amplification due to the catalytic nature and speed of the electron-transfer-triggered depolymerization.

## 2. Results

### 2.1. Polymers

The selected disulfide-based polymers, i.e., pDTT, (Me)pDTT, (AQ)pDTT, and (AQ)(Me)pDTT (Scheme 1), were synthesized following previously established protocols with few modifications [29,31]. HTpDTT was synthesized by exchanging the pyridylsulfide end-caps by hexane-1-thiol in a thiol–disulfide exchange reaction. The examined polymers had a degree of polymerization of either 15 or 23. NMR spectra and size exclusion chromatography (SEC) elugrams can be found in the Supplementary Materials (Figures S1–S10), with yields, conversions, and molecular weights summarized in Table S1.

### 2.2. Electrolyzes of Poly(dithiothreitol) Derivatives

In indirect electrolysis of pDTT, HTpDTT, and (Me)pDTT, AQ served as a mediator using a concentration of 3 mM. The applied potential of −1.07 V vs. SCE was determined from a cyclic voltammetric recording of AQ (i.e., 150 mV more negative than the reduction potential of the first wave; Figure S11). In comparison, the (AQ)pDTT and (AQ)(Me)pDTT polymers comprising pendant AQ units had no need for additional AQ in the solution. In these cases, the applied potential (−0.92 V vs. SCE) was determined to be ~150 mV more negative than the peak potential of the first reduction wave of the polymers. The observed potential shift for AQ as a pendant group is due to the stabilizing effect of the ester linkage between the pDTT backbone and AQ. Aliquots for SEC measurements were taken before and after electrolysis to examine and quantify the depolymerization process.

**Figure 1:**
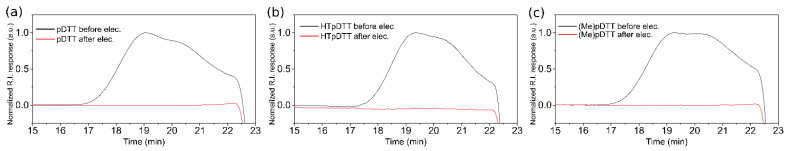
SEC elugrams before (black) and after (red) electrolysis of (**a**) pDTT, (**b**) HTpDTT, and (**c**) (Me)pDTT conducted at −1.07 V vs. SCE with 3 mM AQ in 0.03 M Bu_4_NBF_4_/DMF. Electrolysis was halted once the consumed charge corresponded to a stoichiometry of 3 electrons per polymer chain. Note that these elugrams differ from those for pure polymers in the Supplementary Materials due to presence of AQ and supporting electrolyte.

**Figure 2:**
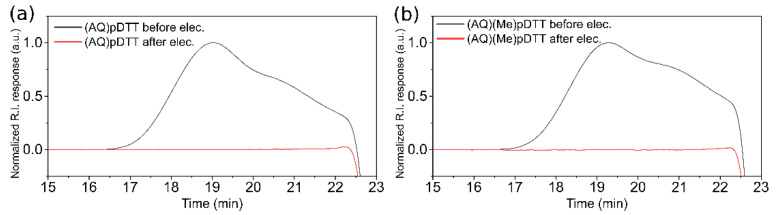
SEC elugrams before (black) and after (red) electrolysis of (**a**) (AQ)pDTT and (**b**) (AQ)(Me)pDTT pDTT conducted at −0.92 V vs. SCE in 0.03 M Bu_4_NBF_4_/DMF. Electrolysis was halted once the charge consumed corresponded to a stoichiometry of 3 electrons per polymer. Note that these elugrams differ from those of pure polymers in the Supplementary Materials due to presence of supporting electrolyte.

Figure 1 and Figure 2 show the elugrams recorded before and after electrolysis for the five polymers. The electrolysis was halted once the consumed charge corresponded to ~three electrons per polymer chain. As observed, all polymers are susceptible to complete depolymerization under these conditions. Prior to electrolysis, a bimodal signal with peaks at 19 and 20.5 min signal is present in the elugrams. After electrolysis, the signal strength in the region of 17–22.5 min is zero; i.e., all the polymer is degraded into small constituents.

GC–MS analyses of depolymerization solutions for pDTT and (Me)pDTT, together with benzyl acetate as an internal standard (1 μL/mL), are available in the Supplementary Materials (Figures S12 and S13). Note that the pDTT solution was derivatized with aceticanhydride prior to GC-MS analyses to mask the hydroxyl groups. The analyses show for both polymers that six-membered disulfide rings, produced from cyclization reactions, are the main product of the electroinduced depolymerization. 

**Figure 3:**
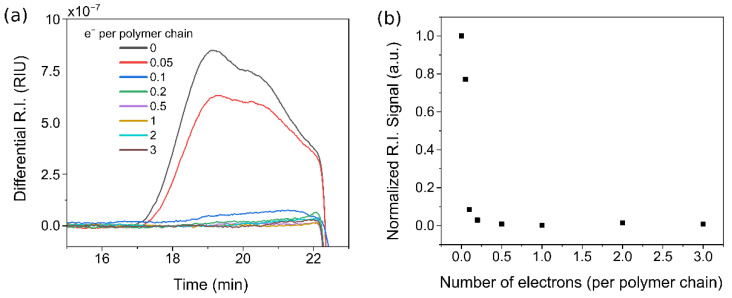
(**a**) SEC elugrams of electrolyzed solution of pDTT in 0.1 M Bu_4_NBF_4_/DMF after consumption of a specified amount of charge expressed as the number of electrons consumed per polymer chain. (**b**) Normalized integral of pDTT SEC elugrams (16.5–22.2 min) vs. the number of electrons consumed relative to the same integral recorded before electrolysis.

Next, depolymerization of DTT was studied in greater detail by collecting aliquots for SEC measurements after passing specified numbers of equivalents of electrons per polymer chain via AQ, going from zero (no depolymerization) to three (complete depolymerization). Figure 3a shows the resulting elugrams, and Figure 3b displays the normalized integrals from the elugrams, in the range 16.5–22.2 min, as a function of the number of electrons used per polymer chain. Surprisingly, a significant decrease in the polymer signal (at 17–22 min) is observable already after consumption of 0.05 electrons per polymer chain, corresponding to ~22% degradation. After consumption of 0.1 electrons per chain, the degradation amounts to 90%, and, beyond 0.1 electrons per polymer chain, complete degradation is accomplished.

To test if this highly efficient electrochemically induced degradation would apply to the remaining polymers, HTpDTT, (Me)pDTT, (AQ)pDTT, and (AQ)(Me)pDTT were all exposed to indirect electrolysis via AQ (or pendant AQ) using 0.5 electrons per polymer chain (Figures S14–S17). Encouragingly, (Me)pDTT and HTpDTT (by and large) are fully depolymerized, while (AQ)pDTT and (AQ)(Me)pDTT are left with some polymer signal. However, the shift to a longer elution time range (19–22 min) is consistent with a significant shortening of the polymer chains. This points to a catalytic process as the remaining signal is less than half of the original one.

### 2.3. Time-Resolved Titration of AQ•^−^ with Polymer Solution

To determine the optimal wavelength for tracking AQ•^−^, a UV–Vis spectrum was recorded of a solution containing electrogenerated AQ•^−^ (Figure S18). This shows an absorption maximum at 556 nm, consistent with the literature value [40]. Using the known extinction coefficient of AQ•^−^ at this wavelength (= 1.1 × 10^4^ M^−1^ cm^−1^ [40]) allows us to convert absorption to concentration of AQ•^−^ and follow the latter with good time resolution.

**Figure 4:**
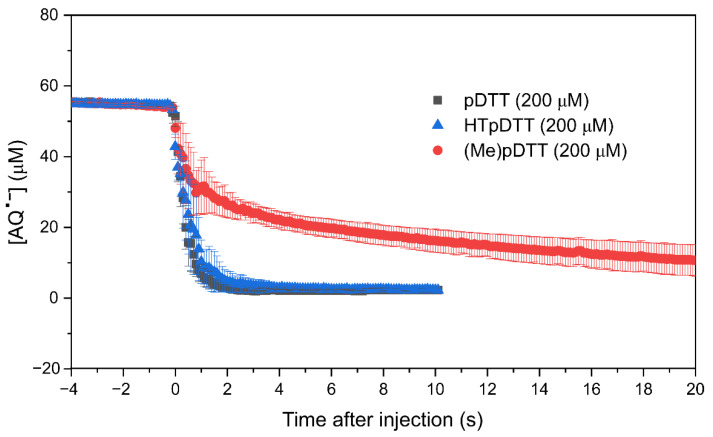
Traces showing change in [AQ•^−^] with time upon adding 100 μL polymer solution (at time = 0 s), resulting in 200 μM polymer concentrations (pDTT black, HTpDTT blue, (Me)pDTT red) in 0.1 M Bu_4_NBF_4_/DMF. Uncertainties are calculated using triplicates.

Figure 4 shows the change in [AQ•^−^] before and after the addition of polymers. First, 55 μM AQ•^−^ was electrogenerated by applying an electrolysis potential of −1.07 V. The stability of AQ•^−^ is observed to be relatively good until a degassed solution of pDTT, HTpDTT, or (Me)pDTT (200 μM resulting concentration) is added (defined as time zero). For pDTT and HTpDTT, AQ•^−^ is consumed within 1–2 s, while, for (Me)pDTT, a sharp decrease in [AQ•^−^] to ~25 μM is detected within seconds, whereafter a slow decrease is observed, reaching ~15 μM after 20 s and zero after 70 s (Figure S19).

### 2.4. Cyclic Voltammetry of pDTT Derivatives, Model Compounds, and AQ

**Figure 5:**
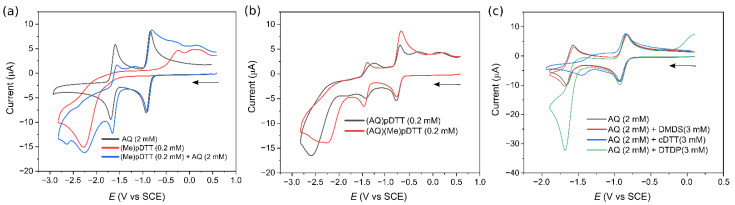
Cyclic voltammograms recorded at a 1 mm GC disk electrode using a sweep rate of 1 V s^−1^ on (**a**) AQ (black), (Me)pDTT (red), and AQ/(Me)pDTT (blue), (**b**) (AQ)pDTT (black) and (AQ)(Me)pDTT (red), and (**c**) AQ (black), AQ/DMDS (red), AQ/cDTT (blue), and AQ/DTDP (green) in 0.1 M Bu_4_NBF_4_/DMF. Arrows indicate scan direction from the resting potential.

Figure 5 displays a collection of cyclic voltammograms of pDTT derivatives without and with AQ present in 0.1 M Bu_4_NBF_4_/DMF. Included are also voltammograms of three model compounds in terms of 1,2-dithiane-4,5-diol (cDTT), dimethyldisulfide (DMDS), and 2,2′-dithiodipyridine (DTDP) in the presence of AQ. These compounds nicely correspond to or mimic the end product, the backbone, and the end-caps of the polymers.

Reduction of AQ takes place as two Nernstian one-electron transfer processes at −0.9 V vs. SCE (AQ to AQ•^−^) and −1.7 V vs. SCE (AQ•^−^ to AQ^2−^), respectively (Figure 5a). The voltammogram of (Me)pDTT shows during the cathodic sweep a small shoulder at −1.8 V and a large irreversible peak at −2.2 V vs. SCE, attributed to the reduction of dialkyldisulfide bonds (Figure 5a). On the anodic sweep, two oxidation peaks are present at −0.2 and 0.2 V vs. SCE, respectively, attributed to the oxidation of thiolates. Upon adding AQ to the (Me)pDTT solution, the first reduction wave observed at −0.9 V (AQ to AQ•^−^) still appears Nernstian. In contrast, the second wave at −1.7 V (AQ•^−^ to AQ^2−^) exhibits a noticeable increase in the peak current, coming along with a diminishing current of the oxidation wave at −1.5 V (AQ^2−^ to AQ•^−^) on the anodic sweep. The anodic peak at −0.8 V (AQ•^−^ to AQ) is of similar size as before but begins to overlap with the oxidation of the thiolates at −0.2 V. This would suggest that transfer of electrons to (Me)pDTT from the more potent electron donor, AQ^2−^, can take place, while AQ•^−^ has no such ability on the time scale of a voltammetric cycle. Furthermore, the additional reduction peaks observed at −2.7 V indicate that, besides direct reduction of the polymer itself, other disulfide-containing species, originating from the depolymerization, may contribute to these signals.

Cyclic voltammograms of pDTT and HTpDTT with and without AQ can be found in the Supplementary Materials (Figures S20 and S21). pDTT exhibits a disulfide reduction peak at −2.2 V. In the presence of AQ, the reduction at −0.9 V vs. SCE (AQ to AQ•^−^) looks similar to that of AQ alone, while the second reduction wave comprising two peaks shifts in a positive direction. The reduction wave of the polymer itself shifts towards a more negative potential and becomes larger in the presence of AQ. For HTpDTT the electron transfer pertaining to the first reduction peak at −0.9 V (AQ to AQ•^−^) is Nernstian, while the second reduction peak shifts to a less negative potential. The reduction peak for the polymer itself shifts to a more negative potential (−2.7 V vs. SCE) and becomes broader, but with little change to the peak current.

For (AQ)pDTT and (AQ)(Me)pDTT, it may be noted that the two reduction peaks for the AQ moiety are shifted by ~150 mV compared with AQ itself. This shift is explained by the charge stabilization effect exerted by the ester linkage. The broad feature of the first reduction wave for (AQ)pDTT is due to the remaining 81% pendant hydroxyl groups (see Scheme 1), which act as proton donors, giving rise to protonation and some disproportionation of AQ•^−^ to AQ and dihydroanthraquinone [41,42]. In addition, the reduction peak of the disulfides is shifted in a negative direction to −2.6 V.

Figure 5c shows voltammograms of AQ by itself and together with DMDS, cDTT, and DTDP. In the presence of DMDS, the first Nernstian one-electron transfer process at −0.9 V vs. SCE (AQ to AQ•^−^) is unaltered, while a slight current increase is observed for the second reduction peak at −1.7 V vs. SCE (AQ•^−^ to AQ^2−^). In comparison, the voltammogram of AQ/cDTT shows many of the same features, although with a broadening and shift of the second reduction peak in a positive direction and a missing oxidation wave for AQ^2−^ to AQ•^−^. From this, we conclude that electron transfer from either AQ•^−^ or AQ^2−^ to simple disulfide bonds is quite slow and that cDTT may act as a proton donor towards, in particular, AQ^2−^. In comparison, a slight increase in peak current is observed for AQ/DTDP already at the first reduction peak at −0.9 V, while the second reduction peak at −1.7 V experiences a 3.5-fold increase with no oxidation peak appearing at −1.5 V on the reverse sweep. This indicates that the electron transfer to the more easily reduced DTDP occurs relatively fast as it is even detectable in the time window of cyclic voltammetry.

Additional voltammograms recorded while increasing concentrations of the model compounds are available in the Supplementary Materials (Figures S22–S24). In general, they show a more profound effect on the two reduction waves due to the increase in the electron transfer rates as a result of higher substrate concentrations. In particular, for cDTT, the voltammograms are going from two separate one-electron processes to a single two-electron process. Based on a comparison of the current increase in the second reduction wave with simulated curves using DigiSim^®^ (assuming an EEC’ mechanism), we were able to extract the rate constant of electron transfer (*k*) for AQ^2−^ to DMDS, DTDP, and (Me)pDTT (Figures S25–S27) [43], with *k*_DMDS_ = 2 × 10^2^ M^−1^ s^−1^, *k*_DTDP_ = 10^5^ M^−1^ s^−1^, and *k*_(Me)pDTT_ = 3 × 10^3^ M^−1^ s^−1^. The corresponding rate constants of electron transfer from AQ•^−^ could not be accurately determined by cyclic voltammetry. However, according to simulations, they can be at most 10^2^ M^−1^ s^−1^ and 3 × 10^2^ M^−1^ s^−1^ for DMDS and DTDP, respectively (Figures S28 and S29). Most likely, they are significantly smaller than these values as AQ•^−^ compared with AQ^2−^ is a 0.8 eV less potent electron donor according to the reduction potentials recorded in cyclic voltammetry.

## 3. Discussion

### 3.1. Depolymerization of pDTT Derivatives Using Redox Catalysis

Electrolysis and titration results show that the indirect reduction, and, thus, depolymerization, of pDTT derivates using AQ•^−^ as an electron donor is feasible. Although no current increase is observed at the first wave in the cyclic voltammograms of AQ/polymer, this can be explained by the different time scales used, with voltammetric sweeping occurring within seconds and electrolyzes in the range of minutes. In line with this interpretation is the observation that it is possible to induce changes to the first reduction wave of AQ using higher substrate concentrations of the model compounds. As expected, the effect on the second wave pertaining to AQ•^−^/AQ^2−^ (located at a 0.8 V potential more negative than the first reduction potential) is more profound. Earlier studies on indirect reduction of simple disulfides demonstrated that electrogenerated AQ•^−^ can be used to reduce disulfides to thiolates on a time scale of min/h [36,38].

Likewise, the intramolecular reduction of disulfide bonds in (AQ)pDTT, using the pendant AQ groups as a mediated electron carrier, show full depolymerization [29], although no visible effect can be seen on the timescale of voltammetry. One of the great advantages of conducting electrolysis of pDTT and its derivatives using AQ as a mediator is that it enables us to induce depolymerization at its first-wave potential of −1.07 V (for the intramolecular case even at −0.92 V) vs. SCE, corresponding to a >1 V potential gain compared with the direct reduction of disulfide bonds taking place at around −2.2 V vs. SCE. In addition, electrolyzes of the AQ/polymers using three equivalents of electrons show full depolymerization of all samples, as evidenced by the complete disappearance of the signal in the SEC elugrams in the region of 17–22 min.

### 3.2. Catalytic Depolymerization

Depolymerization using AQ in a mediated electron transfer has a catalytic component, with as little as 0.2 electrons used per polymer chain to accomplish complete depolymerization of pDTT (Figure 3). Non-catalytic SIP depolymerization through end-cap removal by disulfide bond cleavage would be expected to require two electrons per polymer chain, with one electron going to disulfide bond cleavage and a second to reduce the thus formed thiyl radical to thiolate. However, electrolyzes of the remaining polymers using ½ electron per polymer chain (Figures S14–S17) result in (Me)pDTT being fully depolymerized, while HTpDTT reaches 94% depolymerization; (AQ)pDTT ends at 86% and (AQ)(Me)pDTT at 73%, as calculated using the integrals of the SEC elugrams before and after electrolysis. For (AQ)pDTT and (AQ)(Me)pDTT, a significant signal from polymeric material is still left in the SEC elugrams. Yet, the degree of depolymerization exceeds the 50% that corresponds to the theoretical maximum of an intramolecular SIP degradation with one disulfide bond being reduced by one electron per chain.

The feature that (AQ)pDTT and (AQ)(Me)pDTT do not fully depolymerize under these conditions could be due to the +150 mV shift in reduction potential of the pendant AQ compared with that of AQ itself. The consequence of lowering the driving force is a decrease in the rate of electron transfer to the disulfide bonds in the polymers. Based on these results, we conclude that AQ enables electrocatalytic depolymerization of all pDTT derivatives. Furthermore, this can be accomplished both inter- and intramolecularly, but, with (AQ)pDTT and (AQ)(Me)pDTT, more electrons are required for full depolymerization.

### 3.3. Protonation Pathway

Three of the polymers [pDTT, HTpDTT, and (AQ)pDTT], together with cDTT, contain hydroxyl groups, which may protonate AQ•^−^, let alone AQ^2−^ if generated electrochemically [44]. Both the cyclic voltammetric and time-resolved titration experiments provide evidence of the significant effect the hydroxyl groups are exerting on the reduction processes of AQ. It may be noted that the reduction peak of AQ•^−^ becomes less negative while broadening. At the same time, the second reduction peak completely disappears for the high-concentration experiments of cDTT (Figure S24), which does not occur for DMDS containing no hydroxyl groups (Figure S22). In this respect, the comparison of traces of AQ•^−^ (starting with 55 μM) upon adding either pDTT or (Me)pDTT (in 200 μM) is interesting (Figure 4) as all hydroxyl groups in the latter polymer are acetylated to impede the protonation pathway. While the consumption of AQ•^−^ amounts to 30 μM within the first couple of seconds for (Me)pDTT, all 55 μM AQ•^–^ are used in the case of pDTT because of the competing protonation process. For (Me)pDTT, it is reasonable to assume that all electrons go exclusively to the reduction of disulfide bonds, corresponding to ~0.15 electrons per polymer chain. Despite the presence of the protonation process in the case of pDTT, depolymerization is still effective. According to the titration experiment in Figure 3 (based on SEC analysis), a consumption of 0.2–0.5 electrons per polymer chain is sufficient for accomplishing full depolymerization.

### 3.4. Effect of End-Cap

Regarding the three model compounds, both DMDS and cDTT comprise aliphatic disulfide bonds, thus mimicking the end-caps of HTpDTT as well as the dialkyl disulfide bonds along the backbone of all polymers. Note that cDTT also contains hydroxyl groups and is cyclic, yet without ring tension. Likewise, DTDP is meant to serve as a model compound mimicking pyridyl disulfide end-caps. Acknowledging that the four polymers, pDTT, (Me)pDTT, (AQ)pDTT, and (AQ)(Me)pDTT, contain a mixed alkyl pyridyl disulfide end-cap, the reduction potential of this end-cap should thus be located somewhere between those of DMDS and DTDP.

The cyclic voltammograms (Figure 5) reveal that electron transfer to DTDP is much faster than to DMDS by a factor of ~500, as calculated from the determined rate constants (*k*_DMDS_ = 2 × 10^2^ M^−1^ s^−1^, *k*_DTDP_ = 10^5^ M^−1^ s^−1^), because of a greater extent of electron delocalization onto the pyridyl groups. This decreases significantly the reorganization energy of the stepwise dissociative electron transfer [38,39]. Both AQ•^−^ and AQ^2−^ are capable of transferring electrons to the disulfide bond in DTDP, although AQ^2−^ is a significantly more potent electron donor, enjoying a 0.8 eV greater driving force. All polymers, with the exception of HTpDTT, are characterized by having an alkylpyridyl disulfide end-cap, which should be more easily reduced than the corresponding dialkyl disulfide moiety in HTpDTT and DMDS but more difficult than dipyridyl disulfide in DTDP. This is confirmed by the extracted rate constants of electrons transfer, which follows the order DTDP > (Me)pDTT > DMDS (*k*_(Me)pDTT_ = 3 × 10^3^ M^−1^ s^−1^; measured for AQ^2−^). The titration experiments substantiate this conclusion as both pDTT (Figure 3) and (Me)pDTT (Figure 4 and Figure S15), but not HTpDTT (Figure S14), experience complete depolymerization using less than ½ electron per polymer chain. Based on these studies, we conclude that the electron transfer is preferentially, but not exclusively, directed toward the thiopyridyl end-caps rather than dialkyl disulfide bonds in the backbone, with HTpDTT showing that electron transfer to such bonds is feasible, albeit slower.

### 3.5. Electrocatalytic Depolymerization Mechanism

We have shown that electrochemical generation of AQ•^−^ followed by depolymerization can be conducted with AQ in solution or with AQ as a pendant group in the polymer. Both the disulfide-based end-caps and disulfide bonds in the backbone can be cleaved by dissociative electron transfer, although the pyridyl disulfide containing end-caps are the easiest to reduce. In addition, depolymerization can occur both with and without hydroxyls present, showing that competing protonation reactions do not pose a serious hindrance, although the electron consumption may increase somewhat. Most importantly, the depolymerization is noticed to be of catalytic nature.

**Scheme 2:**
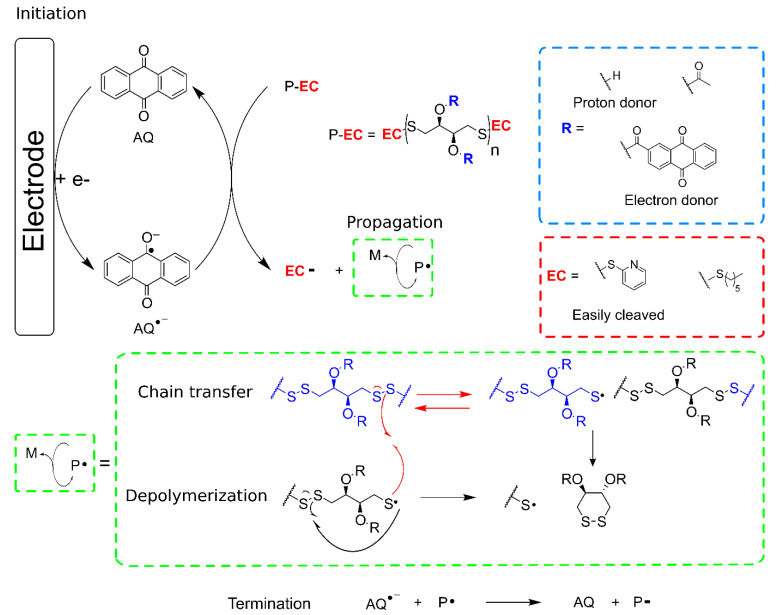
Proposed mechanism for catalytic depolymerization of poly(dithiothreitol) derivatives.

To explain all these features for the electrochemically triggered depolymerization using AQ•^−^, we propose a mechanism where the intermediacy of thiyl radicals plays a crucial role (Scheme 2). According to this hypothesis, thiyl radicals are formed in an initiation step together with thiolates after a mediated dissociative electron transfer to the disulfide moieties (preferentially end-caps). This is followed by a chain reaction involving thiyl radicals reacting with disulfide bonds, both inter- and intramolecularly, allowing for chain transfer of the radical. Each chain reaction will generate a new disulfide bond and thiyl radical and will, as such, only be driven by entropy chopping up the disulfide bonds of the polymer and replacing them with smaller disulfides, ultimately cDTT. This interpretation is supported by earlier research that has shown the easy interconversion between disulfide polymers with short linkages between the disulfide moeities and cyclic small-molecule disulfides [45]. Later, Peron and coworkers estimated the energy barrier of thiyl radical disulfide exchange to be as low as 5 kcal mol^−1^ [46], which would make the chain reaction exceedingly fast. In addition, recent research on disulfide bonds in proteins has shown the importance of disulfide bond scrambling, with radical species appointed as being responsible, when analyzed in mass spectrometry [47,48,49]. Finally, thiyl radicals have been shown to react much faster than corresponding thiolates in self-healing materials [50].

In our case, termination of the chain reaction is suggested to take place once thiyl radicals become further reduced by AQ•^−^ to thiolates. This reduction process is fast, essentially diffusion-controlled, occurring with a rate constant of ~10^9^ M^−1^ s^−1^ [36,38]. However, under our conditions with [AQ•^−^] being in the μM range, the actual rate will be lower, leaving time for the thiyl radicals to first engage in the reaction with disulfide bonds (being in the mM concentration range). Note that direct electrochemical reduction of the disulfide polymer, applying higher concentrations of AQ•^−^ or using a multi-electron donor (e.g., AQ^2−^), would enhance the rate of reducing the thiyl radical to the corresponding thiolate. The result of such inhibition of the chain transfer would be a less catalytic, yet feasible, degradation process.

The involvement of radicals in the depolymerization explains the speed of the degradation, the cross-over of the degradation between polymer chains, and why the reaction is catalytic in the number of electrons consumed to reach complete polymer degradation. With an energy barrier as low as 5 kcal mol^−1^, the thiyl radical–disulfide reaction will be diffusion controlled at room temperatures with a rate constant close to 10^9^ M^−1^ s^−1^. The competition between thiyl radical reacting with disulfide bonds or being further reduced by AQ•^−^ (termination) is, therefore, dependent on the concentration ratio of disulfide bonds and AQ•^−^. With the concentration of the former being in the mM range and the latter in the μM range, the thiyl–disulfide reaction becomes highly favored. This explains the rapid intra- and interpolymer depolymerization (propagation) and why a low charge is sufficient for accomplishing full depolymerization.

The proposed mechanism deviates from the SIP depolymerization of pDTT based on nucleophilic attacks with backbiting thiol–disulfide exchanges. Here, degradation remains intramolecularly in the same polymer chain, and full degradation takes minutes. The normal SIP depolymerization requires stoichiometric amounts of the trigger (DTT) and base (triethylamine) to catalyze the reaction. The faster reaction reported herein will be more favorable to pyridinic end-cap disulfide bonds but can also occur with backbone disulfides. Self-immolative poly(disulfide) materials based on lipoic acid have been shown to depolymerize in an alkaline environment or by addition of reductive thiols, reforming their monomers in high yields [51,52,53]. However, electrochemically induced degradation of self-immolative disulfides, or SIPs in general, is a novel approach, let alone exploitation of its catalytic nature.

For all polymers, triggering appears to be catalytic, with the need for much less than two electrons to achieve full depolymerization. For the intramolecular pathway, higher equivalents of electrons are needed due to slower reactions. Neither the end-cap nor the hydroxyl groups appear to affect the depolymerization to a great extent, which was achievable when either was knocked out. These discoveries open up new avenues for strong signal amplification when less than a stoichiometric amount of reagents can be used to elicit a full response. We envision that this depolymerization method can be employed on any disulfide polymer that would form small cyclic disulfides, i.e., any polymer that would degrade to five- or six-membered disulfide rings, such as poly(lipoic acid) and similar. A path towards better selectivity in the cleavage of end-caps could be a larger potential difference between the end-cap disulfide and the backbone disulfides; i.e., the end-caps should be more readily reduced compared with the backbone. This could be achieved using electron-withdrawing groups, such as nitro or fluorine attached to the aromatic end-cap.

## 4. Materials and Methods

### 4.1. General Materials

Chemicals were purchased from Sigma-Aldrich (Schnelldorf, Germany), with the exception of DTT [IUPAC name: (2*R*,3*R*)-1,4-bis(sulfanyl)butane-2,3-diol/(2S,3S)-1,4-bis(sulfanyl)butane-2,3-diol] from Fischer Scientific (Loughborough, UK) and EDC•HCl from Fluorochem (Hadfield, UK). All chemicals were used without further purification. The polymers pDTT, (Me)pDTT, (AQ)pDTT, and (AQ)(Me)pDTT were synthesized following previously established protocols with a few modifications [29,31]. HTpDTT was synthesized by exchanging the pyridylsulfide end-caps by hexane-1-thiol in a thiol–disulfide exchange reaction; experimental protocols can be found in supporting information. NMR and size exclusion chromatography (SEC) elugrams are available in the Supplementary Materials (Figures S1–S10), with yields, conversions, and molecular weights summarized in Table S1.

### 4.2. General Methods

NMR spectra were recorded using a Bruker 400 MHz spectrometer (Fällanden, Switzerland) with d-DMSO as solvent and as internal reference. SEC was performed using a system comprising an LC-20AD Shimadzu HPLC pump (Kyoto, Japan), a Shimadzu RID-10A refractive index detector (Kyoto, Japan), and a DAWN HELEOS 8 light scattering detector (Dernbach, Germany) from Wyatt. The detector was SPD-M20A PDA, equipped with an Mz-Gel SDplus Linear column (8 × 300 mm) using 5 µm particles from MZ-Analysentechnik to provide an effective molecular weight range of 1 kDa to 1 MDa. *N*,*N*-Dimethylformamide (DMF) containing 10 mM LiBr was employed as solvent. For molar weight calculations, samples were analyzed using a PMMA standard curve. The mechanical mixer used for synthesis of pDTT was a DAC 150.1 FVZ-K SpeedMixer (Hamm, Germany) from Hauschild. GC–MS consisted of a Hewlett-Packard 5890A gaschromatograph (Birkerød, Denmark) equipped with a 5971A MSD. The GC column was an HP5 25 m with 0.25 mm internal diameter, injection temperature was 250 °C, and helium flow 1.0 mL min^−1^. The temperature starting at 80 °C was held for 5 min before being ramped up with 15 °C/min to 230 °C (held for 20 min).

Cyclic voltammetry was recorded with a CH Instrument (601D) potentiostat in a three-necked flask containing 0.1 M Bu_4_NBF_4_/DMF purged with Ar for at least 10 min prior to recordings. As working electrode, a homemade glassy carbon (GC) disc electrode (Sigradur G, diameter = 1 mm, embedded in epoxy resin) was used while a Pt wire served as counter electrode. As quasi reference electrode, an Ag/AgI electrode was employed. Potentials were referred to saturated calomel electrode (SCE) by measuring the redox potential of a 2 mM ferrocene (0.432 V vs. SCE) solution at the end of each experiment. Polymers were used in a concentration of 0.2 mM and AQ with a concentration of 2.0 mM.

Simulations of linear sweep voltammetry were conducted in DigiSim^®^ assuming an EEC’ mechanism for the second reduction wave of AQ with *K*_eq_ = 10^10^ (equilibrium constant for the homogeneous electron transfer), [AQ] = 2 mM, [DMDS]/[DTDP][RSSR] = 5/5/3.2 mM, half peak potentials of AQ locked at −0.87 and −1.61 V vs. SCE at sweep rate = 1 V s^−1^ [43]. Diffusion: semi-infinite, potential step: 0.005 V, and pre-equilibrium: disabled. The value of *k*_f_ (rate of homogenous rate electron transfer) was altered until obtaining the best possible fit between experimental and simulated data, with particular emphasis on size of peak currents. Simulations pertaining to the first reduction wave of AQ werefigur conducted in DigiSim^®^ assuming an EC’ mechanism with *K*_eq_ = 10^10^, [AQ] = 2 mM, [DMDS]/[DTDP] = 24 mM, half peak potential of AQ locked at −0.87 V vs. SCE at sweep rate = 1 V s^−1^ [43]. The value of *k*_f_ was altered until obtaining the best possible fit between experimental and simulated data, with particular emphasis on the size of peak currents.

Electrolysis was conducted using a CH Instrument (601D) potentiostat in a two-necked two-chamber H-cell with 0.03 M Bu_4_NBF_4_/DMF electrolyte separated by a glass frit membrane. A carbon paper electrode was used as working electrode, while a Pt mesh served as counter electrode, and Ag/AgCl as reference electrode. In the first series of electrolyzes, ~3 electrons per polymer chain were passed; volume of catholyte = 5.5 mL, [AQ] = 3 mM, and ~1.5 mg mL^−1^ of polymers were added, providing polymer concentrations for pDTT (0.60 mM), HTpDTT (0.41 mM), (Me)pDTT (0.39 mM), (AQ)pDTT (0.39 mM), and (AQ)(Me)pDTT (0.31 mM) as listed. The catholyte solution was purged with argon for at least 10 min prior to recordings. A cyclic voltammogram was recorded just prior to electrolysis to determine the cathodic peak potential of AQ (or pendant AQ); electrolysis was conducted at a potential 150 mV more negative than this potential and stopped when a specific charge in the range of 0.50–0.96 C was consumed (corresponding to ~3 electrons per polymer chain in each case).

Likewise, electrolyzes with passing of 0.5 electrons per polymer chain were conducted using 1 mg mL^−1^ of polymer, i.e., HTpDTT (0.27 mM), (Me)pDTT (0.26 mM), (AQ)pDTT (0.26 mM), and (AQ)(Me)pDTT (0.21 mM), and stopped after consumption of 0.055–0.072 C. Finally, electrolysis of pDTT passing 0, 0.05, 0.1, 0.2, 0.5, 1.0, 2.0, and 3.0 equiv. of electrons per polymer chain was conducted with pDTT (0.40 mM); volume of catholyte = 15 mL and [AQ] = 3 mM. Aliquots for SEC were collected after the specific amount of charge was consumed.

UV–Vis spectra were collected from the cathode chamber of an H-cell using an Agilent Cary-60 UV–Vis absorption spectrometer with a dip probe from C-Tech with pathlength of 1 cm. The cathodic compartment was filled with 3 mL solution containing 2 mM AQ in 0.1 M Bu_4_NBF_4_/DMF purged with argon. With a dip-in probe inserted in the catholyte, spectra of AQ•^−^ were recorded and compared with those reported in literature [40]. Absorbance of AQ•^−^ was monitored at the wavelength 556 nm with a time resolution of 0.1 s. Lambert–Beer law was used to calculate [AQ•^−^] from the absorbance at 556 nm and by employing $\varepsilon_{556}^{}$ = 11.000 M^−1^ cm^−1^. Solutions of AQ•^−^ were prepared by electrochemical reduction of 2 mM AQ in 0.1 M Bu_4_NBF_4_/DMF. The electrolysis was stopped once the absorbance at 556 nm reached 0.6 corresponding to [AQ•^−^] = 55 μM. While recording the absorbance at 556 nm, 100 μL of a degassed polymer solution of pDTT, HTpDTT, or (Me)pDTT was injected to achieve [polymer] ≈ 0.2 mM. The decay of [AQ•^−^] was traced from this point on, defined as *t* = 0.

## 5. Conclusions

We successfully synthesized poly(dithiothreitol) derivatives by exchanging end-caps and/or introducing various pendant groups via Steglich esterification. All polymers were shown to be depolymerized once triggered by electrogenerated AQ•^−^. Only a catalytic amount of electrons was needed to achieve full depolymerization, which would be in line with an intermolecular chain reaction from a mechanistic point of view. The reaction is rapid (within seconds) and applicable to all derivatives regardless of end-cap and whether they contain an internal proton donor or not. Triggering can be completed using either AQ in solution (intermolecular) or pendant AQ moieties (intramolecular). We propose a mechanism where dissociative electron transfer from AQ•^−^ to a polymer disulfide bond generates a thiyl radical and a thiolate. The former may be engaged in reaction with other disulfide bonds, creating a new disulfide and a thiyl radical, eventually leading to smaller disulfides. The proposed mechanism explains both the catalytic nature and rapidity of these depolymerizations.

## Data Availability

The authors declare data availability by request.

## Supplementary Materials

The following supporting information can be downloaded at: https://www.mdpi.com/article/10.3390/molecules27196292/s1, Additional experimental procedures, NMR spectra, SEC, cycliv voltammograms, and SEC elugrams of the electrolyzes (PDF).

## Sample Availability

Samples of the compounds are available from the authors.
