# Supplementary material for: Electrocatalytic Depolymerization of Self-Immolative Poly(Dithiothreitol) Derivatives
Source: Molecules. 2022 Sep 23;27(19):6292. doi: 10.3390/molecules27196292 (PMC9573698; doi:10.3390/molecules27196292)
Supplement: Supplementary file 1 [file molecules-27-06292-s001.zip › molecules-1915131-supplementary.pdf]

Electrocatalytic Depolymerization of Self-Immolative Poly(dithiothreitol) Derivatives

Magnus Hansen-Felby,[a] Steen Uttrup Pedersen,[a,•] and Kim Daasbjerg.[a,b,•]

[a] Department of Chemistry and Interdisciplinary Nanoscience Center (iNANO), Aarhus University, Langelandsgade 140, DK-8000 Aarhus C, Denmark. [b] Novo Nordisk Foundation CO_2 Research Center, Aarhus University, Gustav Wieds Vej 14, DK-8000 Aarhus C, Denmark.

Materials

All synthetic procedures were carried out under ambient conditions unless otherwise stated. DMF and Bu_4NBF_4 was mixed and dried through a column of Al_2O_3 to remove water. Most solvents and chemicals were purchased from Sigma Aldrich and used without further purification. Dithiothreitol (DTT) was purchased from Fischer Scientific.

Synthesis of pDTT

Poly(dithiothreitol) (pDTT) was synthesized according to a previously reported method [31].^{1}H NMR (400 MHz, d-DMSO) δ 8.49–8.39 (m, 2H), 7.90–7.75 (m, 4H), 7.30–7.18 (m, 2H), 5.41–4.80 (m, 32H), 3.88–3.62 (m, 30H), 3.12–2.60 (m, 72H).

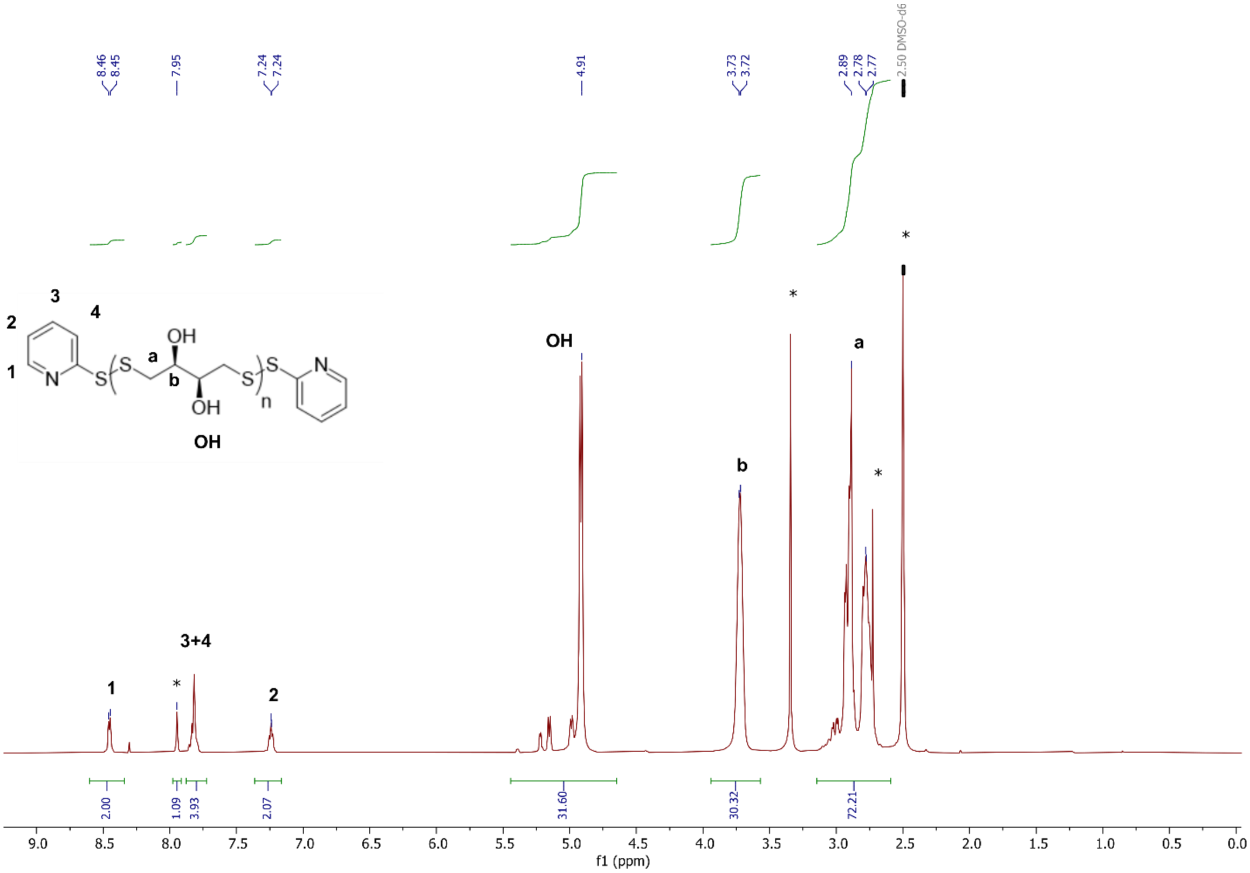

Figure S1. ^{1}H NMR of pDTT (asterisks denotes residual solvent and H_2O peaks).

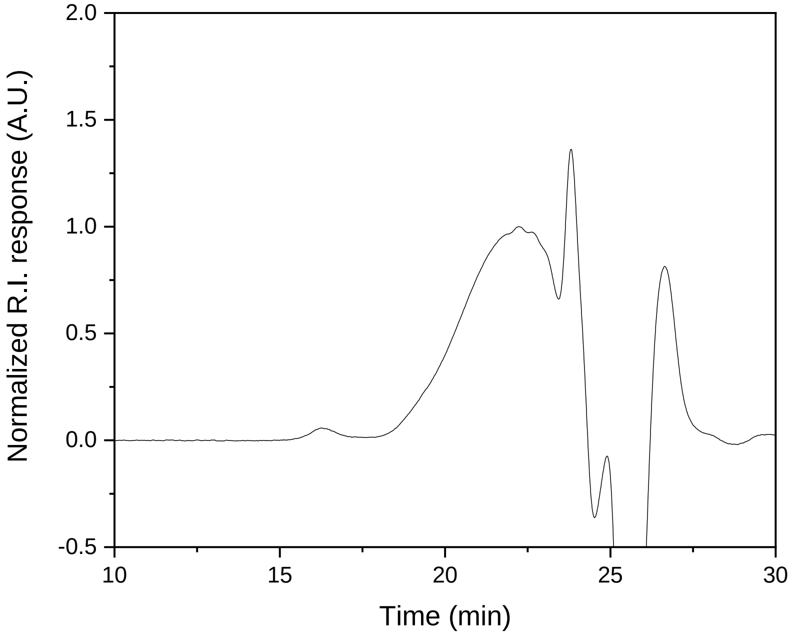

Figure S2. SEC elugram of pDTT normalized to polymer peak at 22 min.

Synthesis of HTpDTT

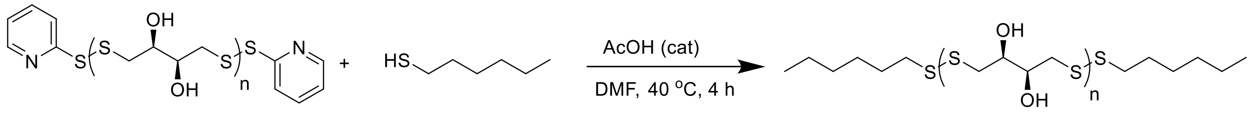

pDTT (200 mg, 40 μmol, 1.0 equiv.) was added to a flame dried test tube equipped with a magnetic stirrer. It was dissolved in DMF (0.8 ml) followed by adding one drop of acetic acid. The solution was degassed with Ar for 10 min while stirring. It was charged with hexanethiol (12 μl, 84 μmol, 2.1 equiv.), and heated to 40 ^{o}C, and allowed to react for 4 h. The solution was precipitated three times into excess pentane:acetone (4:1), which yielded the desired product HTpDTT as a white powder 39 mg (39%). ^{1}H NMR (400 MHz, d-DMSO) δ 4.91 (d, J = 6.3 Hz 46H), 3.73 (bs, 45H), 3.00–2.60 (m, 96H), 2.50 (m, 72H), 1.62 (p, J = 7.4 Hz, 4H), 1.40–1.19 (m, 13H), 0.87 (t, J = 6.3 Hz, 7H).

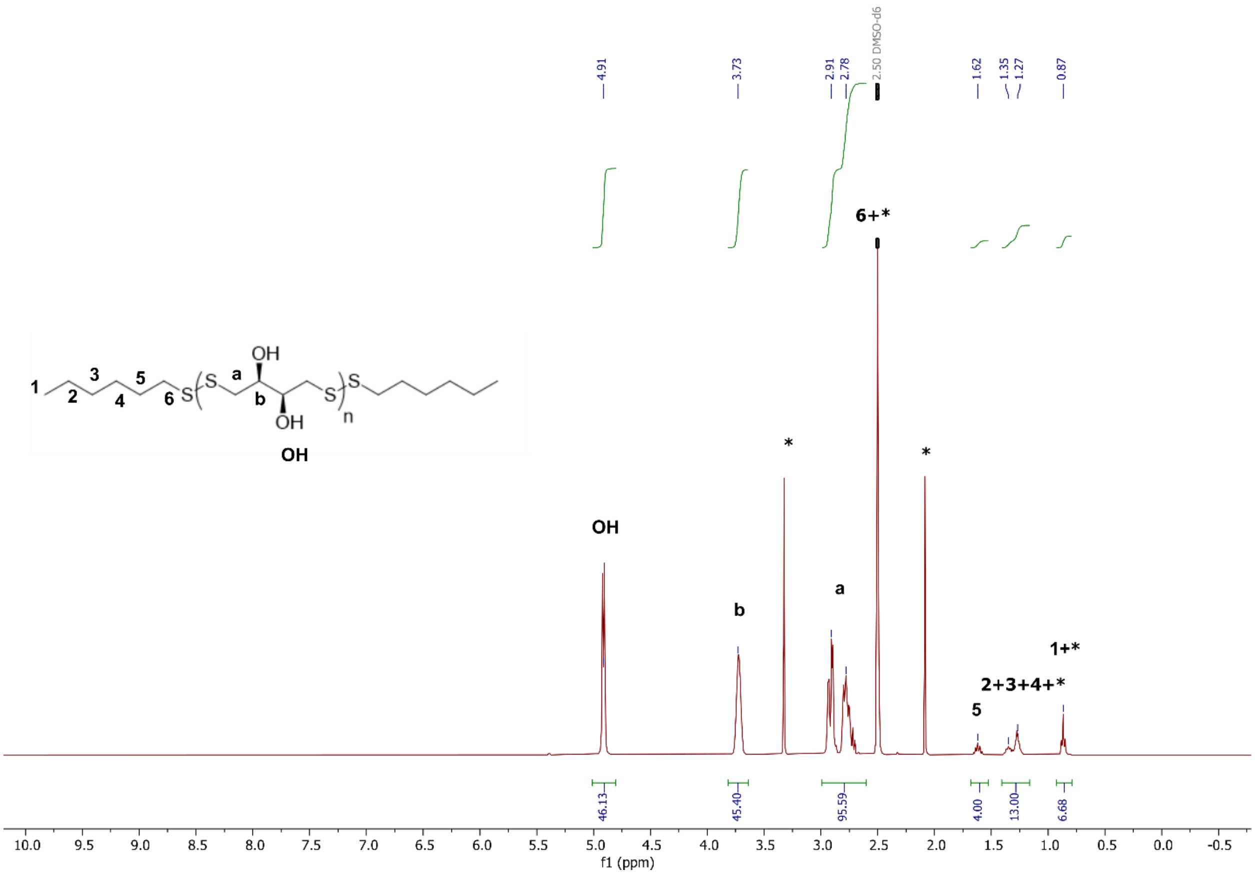

Figure S3. ^{1}H NMR of HTpDTT (asterisks denotes residual solvent and H_2O peaks).

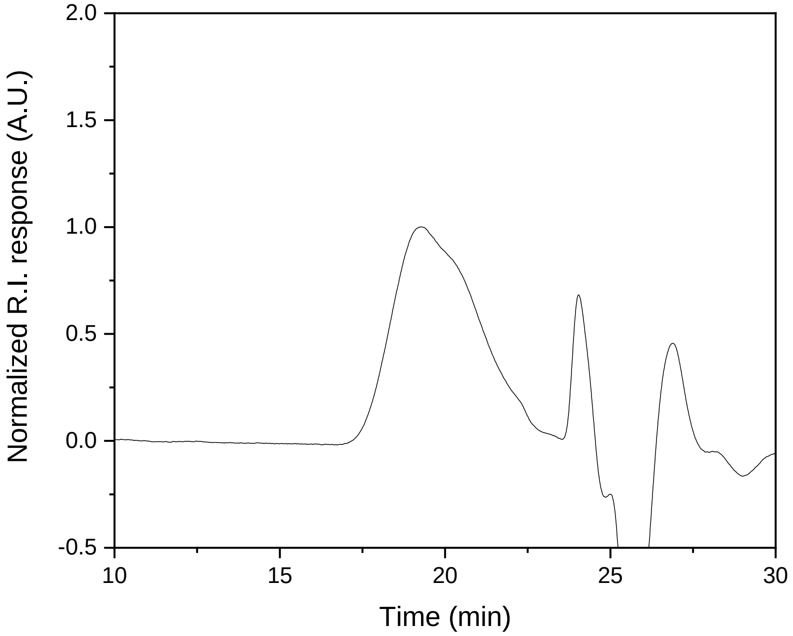

Figure S4. SEC elugram of HTpDTT.

Synthesis of (Me)pDTT

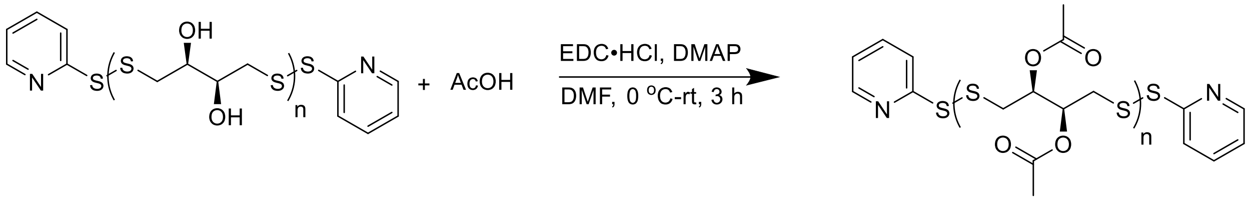

(Me)pDTT was synthesized according to a previously published protocol with modifications [29]. pDTT (200 mg, 2.4 mmol (OH), 1.0 equiv.) was added to a flame dried test tube equipped with a magnetic stirrer. It was dissolved in DMF (1.2 ml) and put under Ar while stirring. The solution was cooled down to 0 °C using an ice bath, followed by addition of EDC•HCl (735 mg, 3.8 mmol, 1.6 equiv.), AcOH (0.22 ml, 3.8 mmol, 1.6 equiv), and DMAP (29 mg, 0.24 mmol, 0.1 equiv.). After 15 min, the ice bath was removed and the solution was allowed to react for 3 h. It was diluted using EtOAc (70 mL), transferred to a separatory funnel and washed twice with water, twice with saturated sodium bicarbonate solution, and once with brine. The organic phase was concentrated under reduced pressure and the resulting white powder was dried in vacuo overnight, yielding the desired product 283 mg (94%). ^{1}H NMR (400 MHz, d-DMSO) δ 8.51–8.44 (m, 2H), 7.89–7.66 (m, 4H), 7.30–7.20 (m, 4H) 5.41–5.18 (m, 29H), 3.13–2.73 (m, 60H), 2.07 (s, 83H).

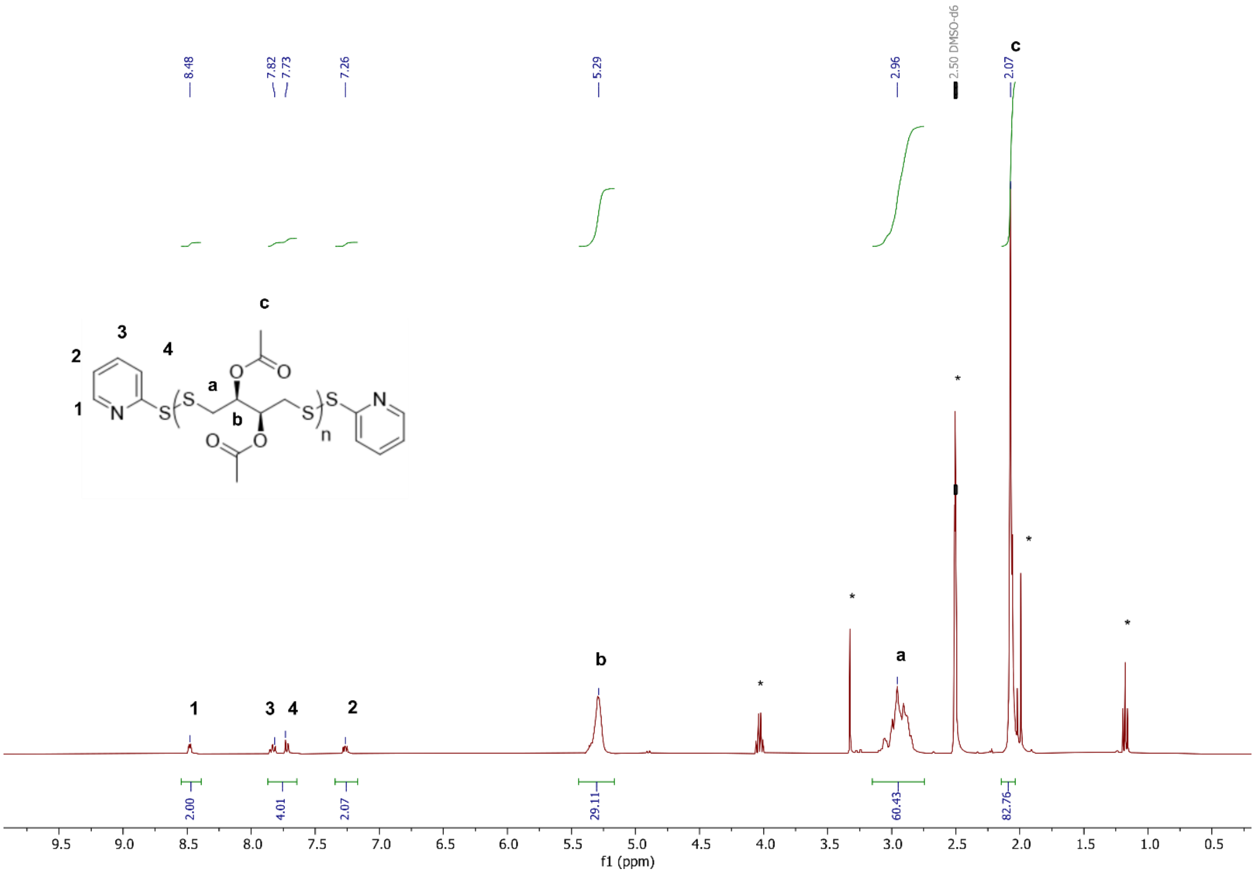

Figure S5. ^{1}H NMR of (Me)pDTT (asterisks denotes residual solvent and H_2O peaks).

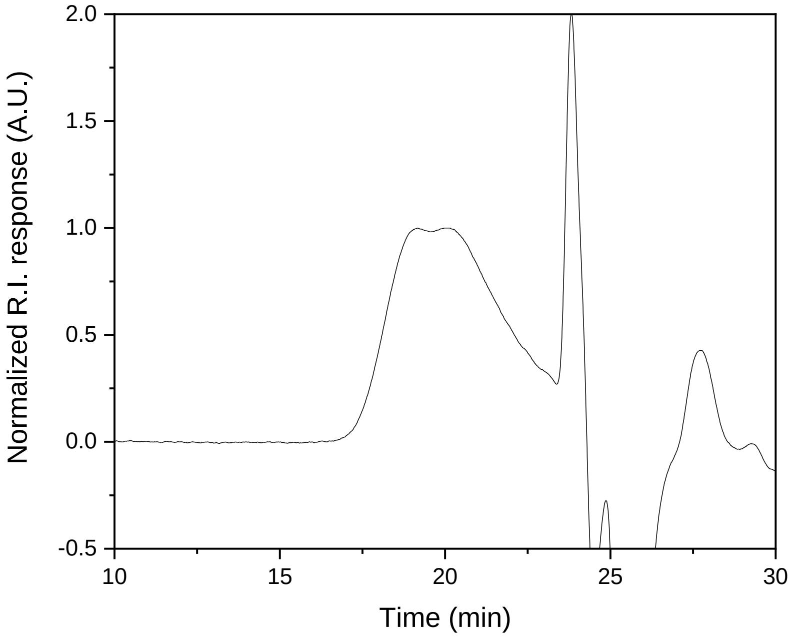

Figure S6. SEC elugram of (Me)pDTT normalized to polymer peak at 20 min.

Synthesis of (AQ)pDTT

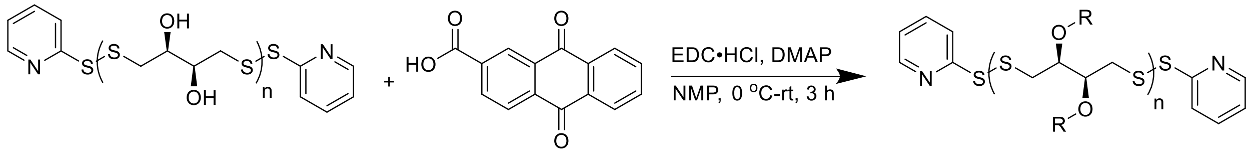

(AQ)pDTT was synthesized according to a previously published protocol with modifications [29]. pDTT (400 mg, 4.8 mmol (OH), 1.0 equiv.) was added to a flame dried 25 ml round bottom flask equipped with a magnetic stirrer. It was dissolved in NMP (10 ml) and put under Ar while stirring. The solution was cooled down to 0 °C using an ice bath, followed by addition of EDC•HCl (1.47 g, 7.7 mmol, 1.6 equiv.). Anthraquinone-2-carboxylic acid (302 mg, 1.2 mmol, 0.25 equiv.), and DMAP (59 mg, 0.48 mmol, 0.1 equiv.). After 15 min, the ice bath was removed and the solution was allowed to react for 3 h. The polymer was precipitated into excess water, the water was decanted off, and the polymer was redissolved in DMSO and precipitated into water. The resulting slightly brown powder was dried in vacuo overnight, yielding the desired product 526 mg (86%). ^{1}H NMR (400 MHz, d-DMSO) δ 8.86–7.64 (m, 55H), 7.27–7.08 (m, 2H), 5.87–5.74 (m, 2.5H), 5.71–5.57 (m, 3H), 5.49–5.33 (m, 3.5 H), 5.19–4.78 (m, 26H), 3.27–2.61 (m, 60H).

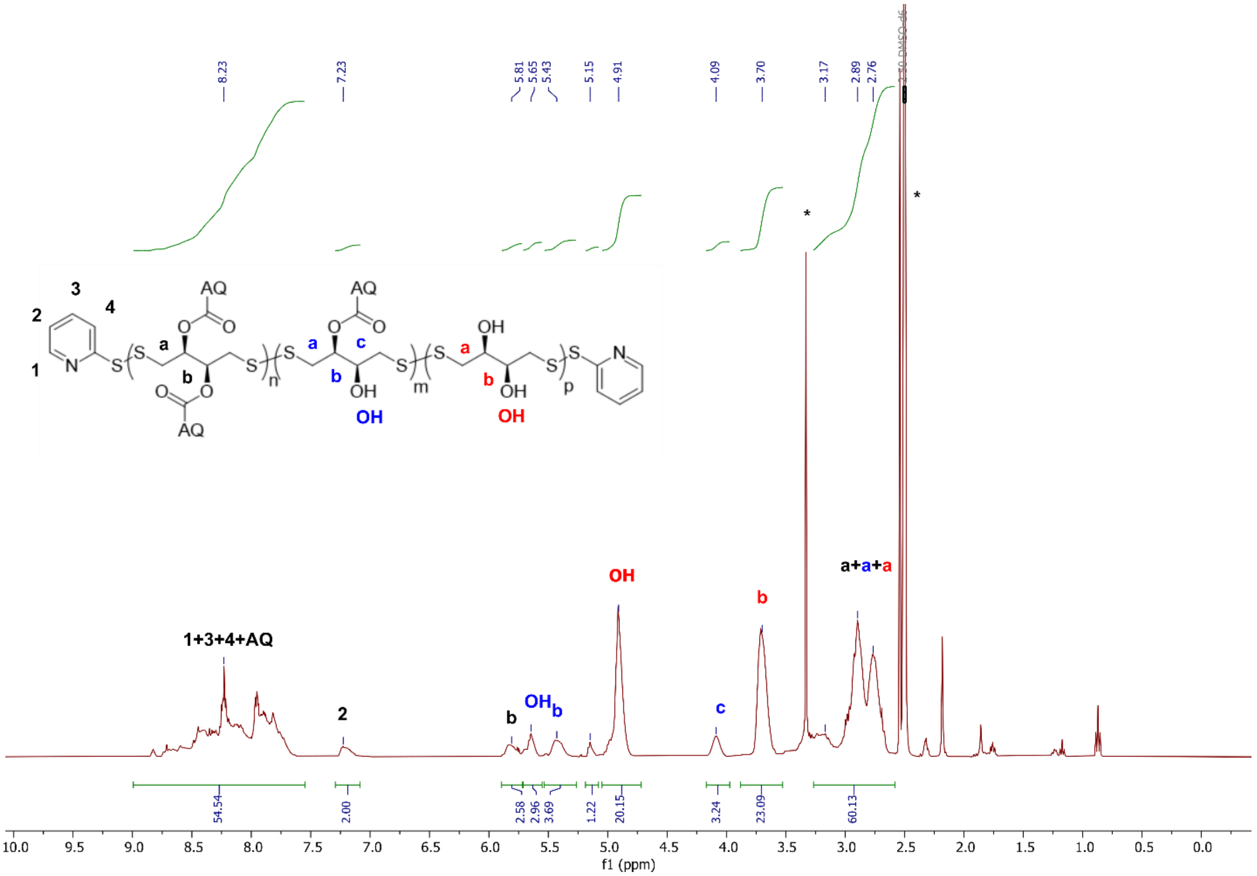

Figure S7. ^{1}H NMR of (AQ)pDTT (asterisks denotes residual solvent and H_2O peaks).

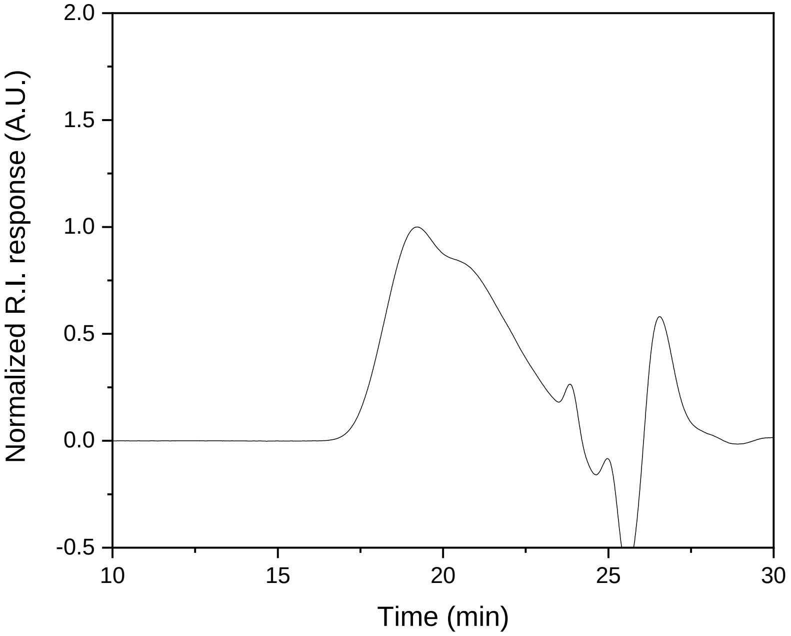

Figure S8. SEC elugram of (AQ)pDTT.

Synthesis of (AQ)(Me)pDTT

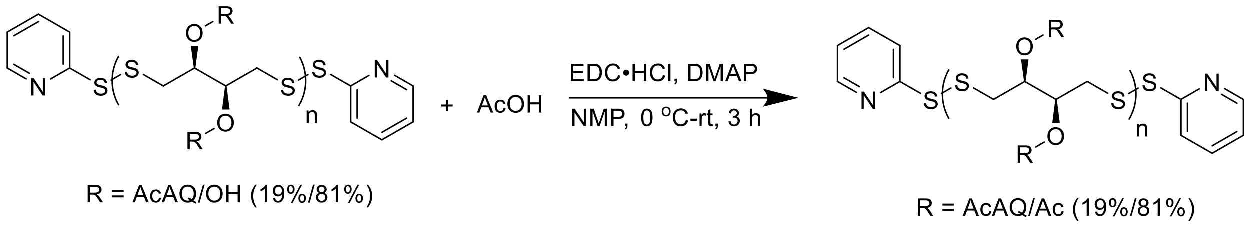

(AQ)pDTT (300 mg, 2.91 mmol (OH), 1.0 equiv. (OH)) was added to a flame dried 25 ml round bottom flask equipped with a magnetic stirrer. It was dissolved in NMP (6 ml) and put under Ar while stirring. EDC•HCl (1.10 g, 5.75 mmol, 1.98 equiv.), AcOH (290 ul, 5.04 mmol, 1.73 equiv.), and DMAP (44 mg, 0.36 mmol, 0.12 equiv.) were added and the solution was allowed to react overnight. It was diluted using EtOAc and transferred to a separatory funnel and washed twice with water, twice with saturated bicarbonate solution, and once with brine. The organic phase was concentrated in vacuo the resulting slightly brown powder was dried in vacuo overnight, yielding the desired product 344 mg, (91%). ^{1}H NMR (400 MHz, d-DMSO) δ 8.74–7.64 (m, 51H), 7.29–7.12 (m, 2H), 5.87–5.72 (m, 2H), 5.71–5.54 (m, 3.5H), 5.54–5.39 (m, 4 H), 5.38–5.16 (m, 21.5H), 3.27–2.58 (m, 59H), 2.05 (bs, 76H)

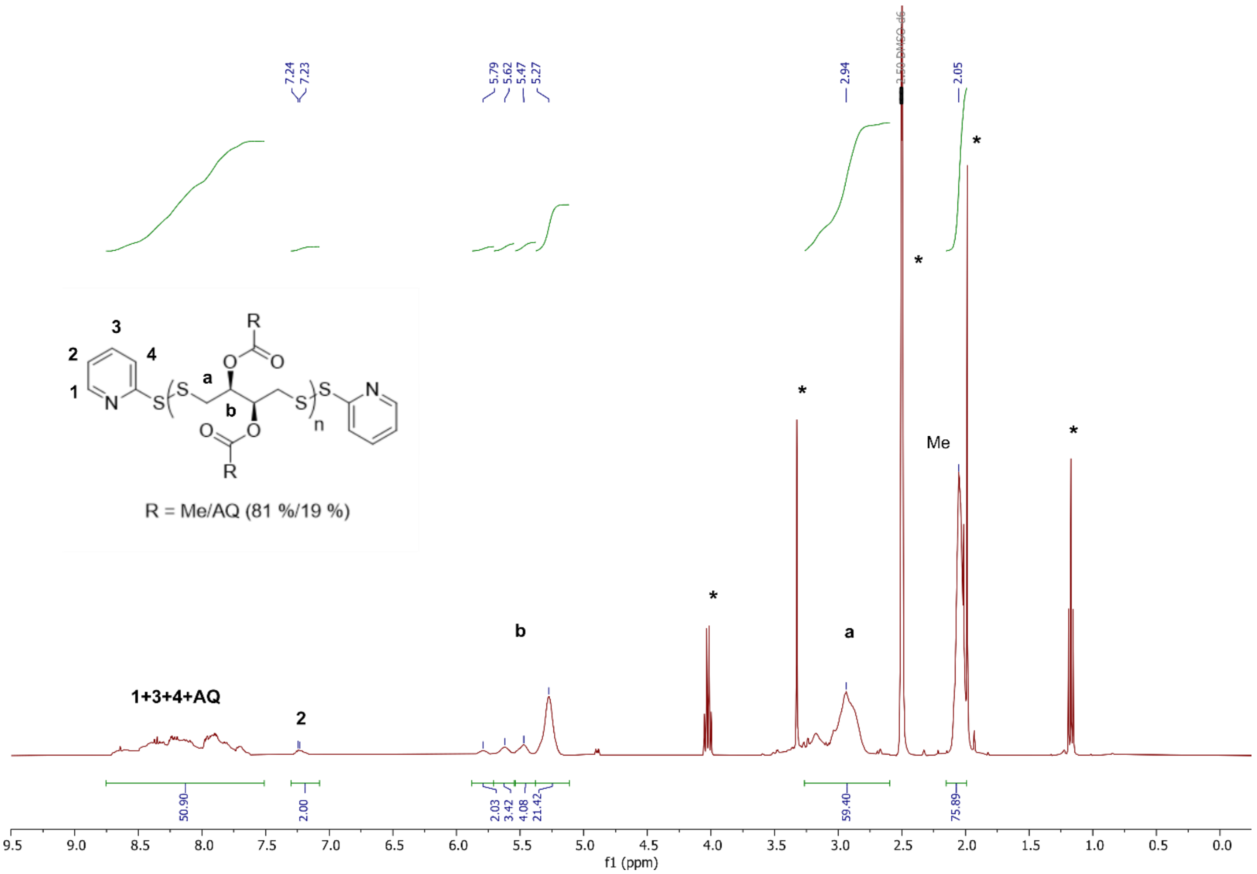

Figure S9. ^1H NMR of (AQ)(Me)pDTT (asterisks denotes residual solvent and H_2O peaks).

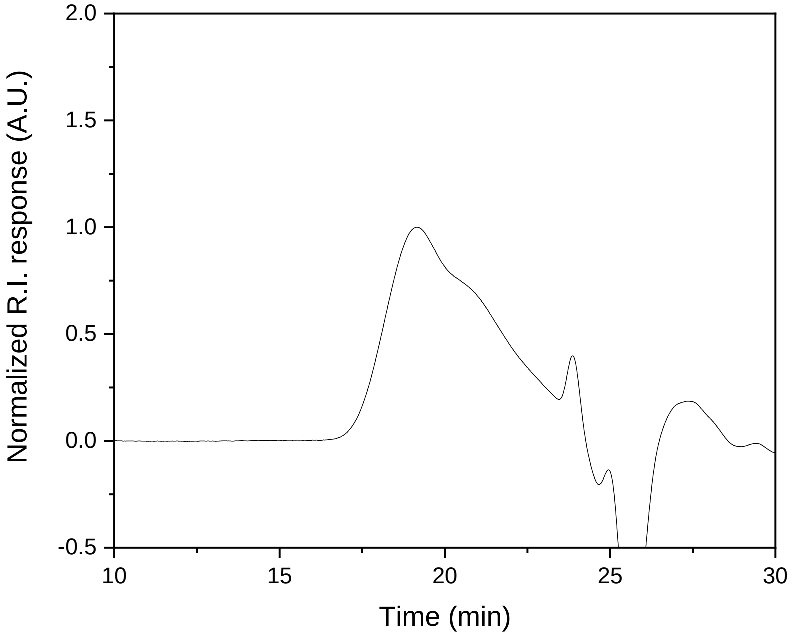

Figure S10. SEC elugram of (AQ)(Me)pDTT.

Molecular weights

Table S1. Conversion, Yields, Molecular Weight, and Polydispersity Index of Poly(dithiothreitol) and Derviatives.

Polymer	Conversion (%)	Yield (%)[a]	DP^b	M_n (kDa)[b]	M_n (kDa)[c]	$Đ^c$
pDTT	--	--	15	2.5	2.4	1.26
HTpDTT	100	39	23	3.7	5.4	1.28
(Me)PDTT	>95	94	15	3.8	4.6	1.42
(AQ)pDTT	19	86	15	3.8	4.6	1.43
(AQ)(Me)pDTT	>95	91	15	4.8	4.7	1.45

[a] Yields calculated as the amount of isolated polymer based on conversion percentage (with conversion >95% assumed to be 100%). [b] Calculated using end group analysis in ^{1}H NMR. [c] Molecular weights and values of $Đ$ determined using size exclusion chromatography (SEC) are reported relative to poly(methyl methacrylate) (PMMA) standards.

Cyclic voltammetry of anthraquinone

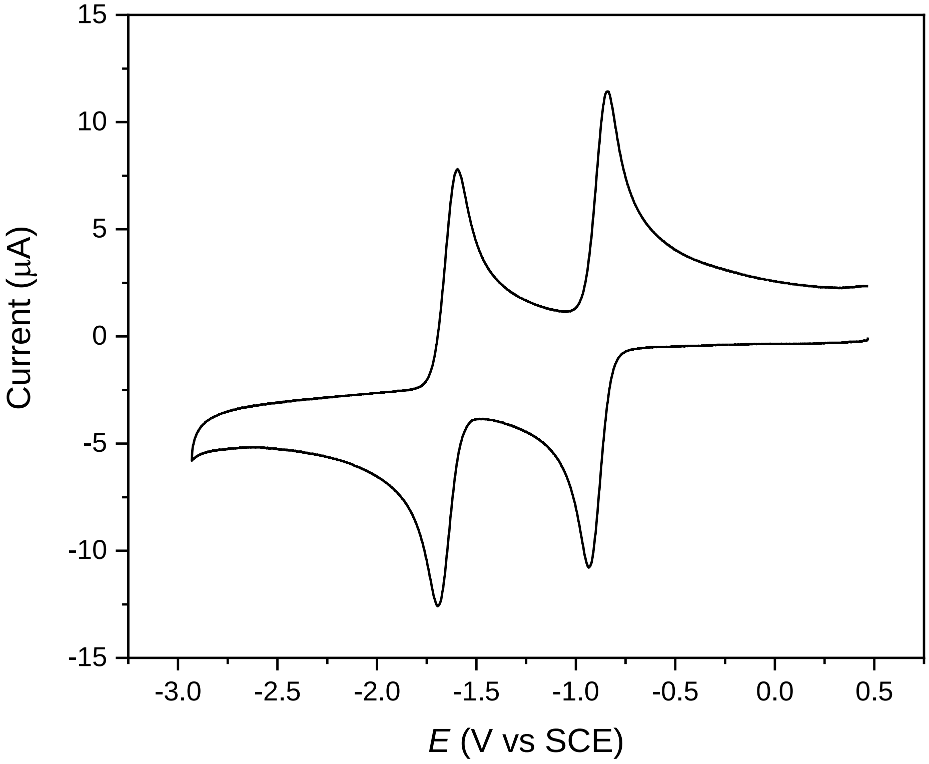

Figure S11. Cyclic voltammograms recorded at a 1 mm GC disk electrode using a sweep rate of 1 V s^{-1} on AQ (2 mM) in 0.1 M Bu_4NBF_4/DMF.

GC-MS of electrolyzed solutions

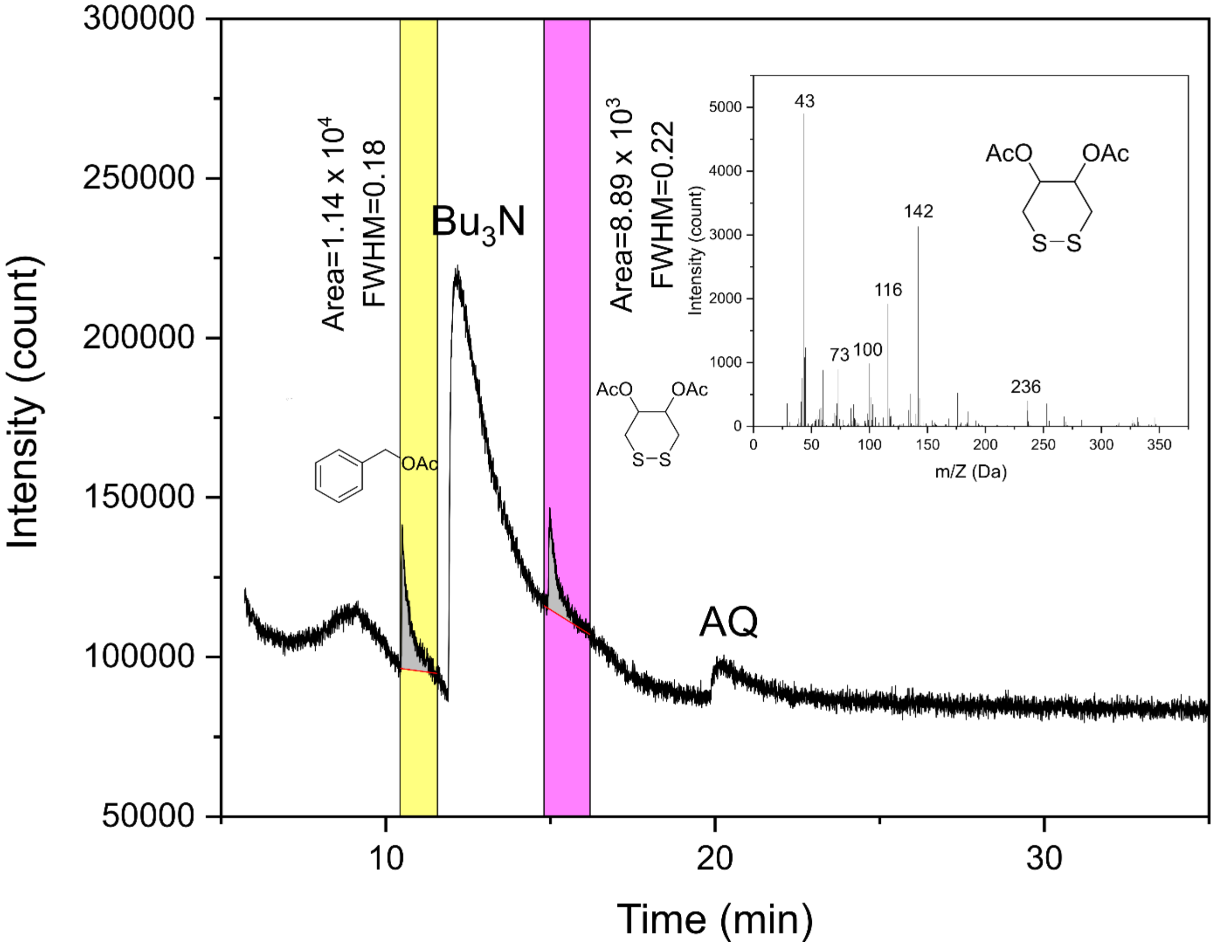

Figure S12. GC-MS chromatogram of pDTT (0.6 mM) electrolysis solution with inset of MS spectrum. Acetic anhydride was added to the solution after electrolysis to functionalize cDTT to make it better detectable in GC-MS. Benzyl acetate was used as internal standard. Bu_3N originates from Hofmann elimination of the supporting electrolyte which deposits in the column over repeated runs.

On the assumption that the degree of polymerization = 15, maximum concentration of cDTT = 9 mM, and concentration of the internal standard benzyl acetate = 7 mM, the amount of cDTT obtained can be calculated to constitute 61% of the expected total amount of depolymerized products. Most likely, the remaining 39% is cDTT as well, but it is not visible in the chromatogram due to not being fully functionalized by acetic anhydride.

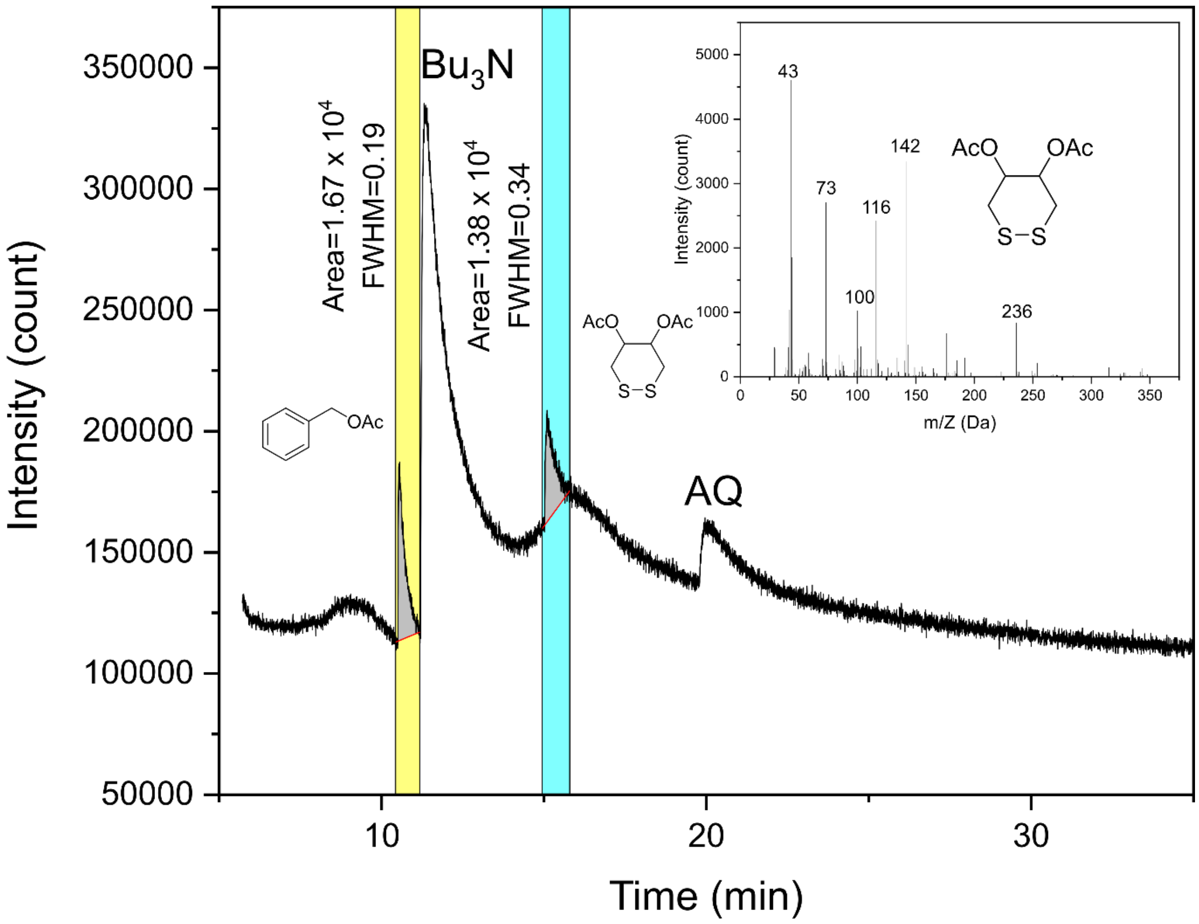

Figure S13. GC-MS chromatogram of (Me)pDTT (0.4 mM) electrolysis solution with inset of MS spectrum. Benzyl acetate was used as internal standard. Bu_3N originates from Hofmann elimination of the supporting electrolyte which deposits in the column over repeated runs.

On the assumption that the degree of polymerization = 15, maximum concentration of (Me)cDTT = 9 mM, and concentration of the internal standard benzyl acetate = 7 mM, the amount of cDTT obtained can be calculated to constitute 97% of the expected total amount of depolymerized products.

Electrolysis using ½ electron per polymer chain

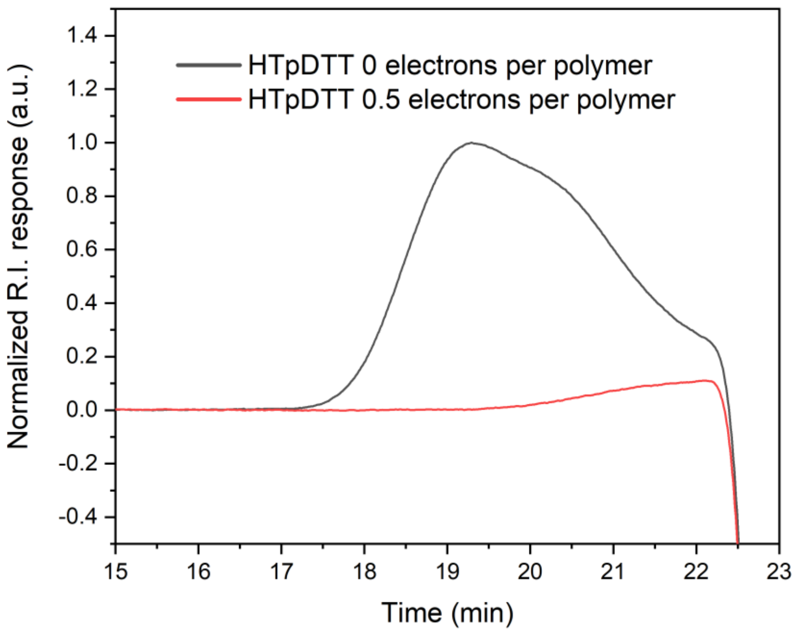

Figure S14. SEC elugrams of HTpDTT before (black) and after (red) electrolysis using ½ electron per polymer chain.

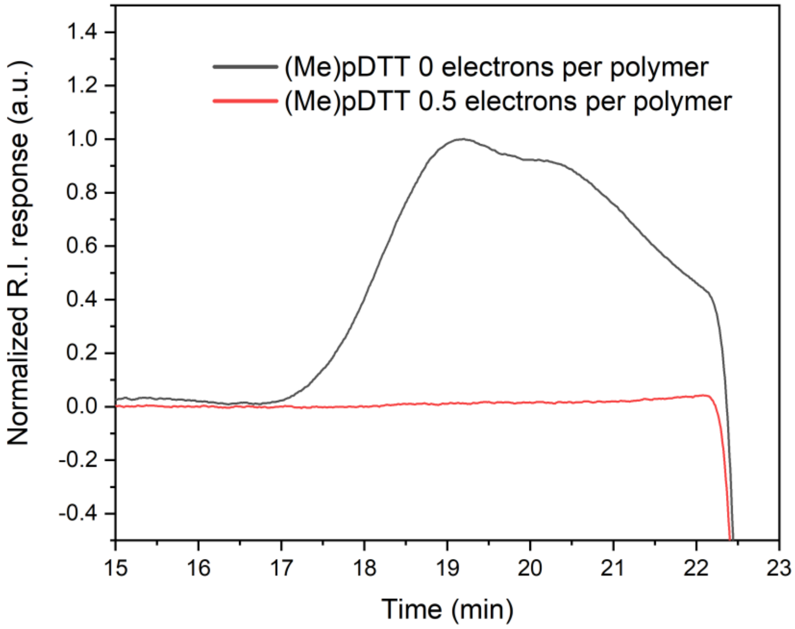

Figure S15. SEC elugrams of (Me)pDTT before (black) and after (red) electrolysis using ½ electron per polymer chain.

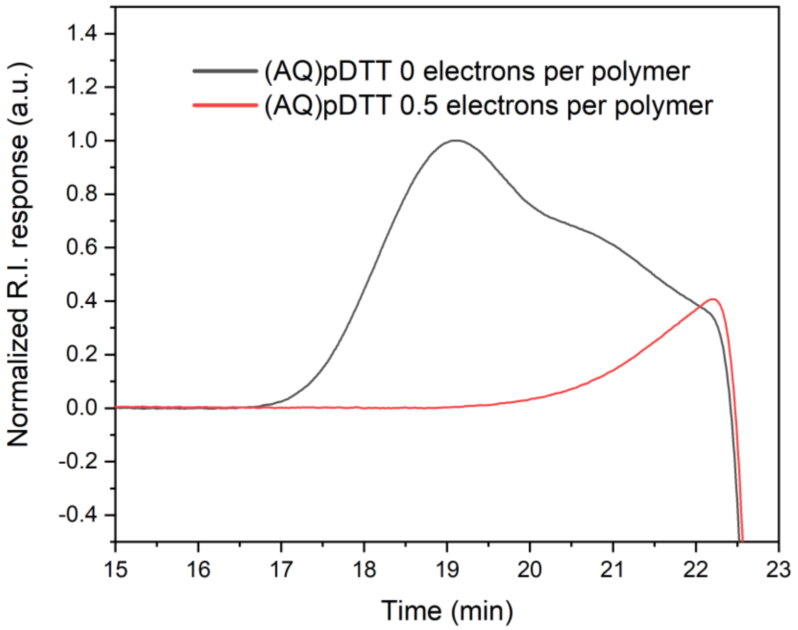

Figure S16. SEC elugrams of (AQ)pDTT before (black) and after (red) electrolysis using ½ electron per polymer chain.

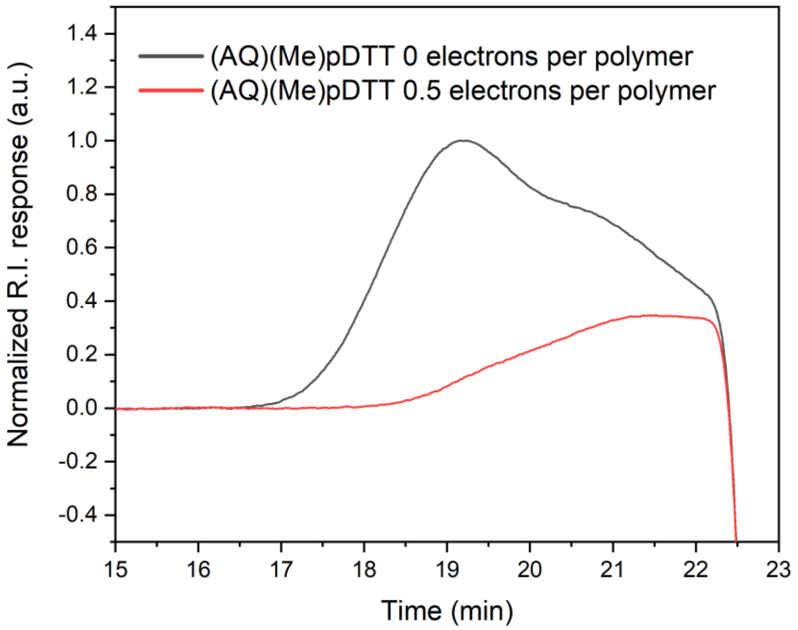

Figure S17. SEC elugrams of (AQ)pDTT before (black) and after (red) electrolysis using ½ electron per polymer chain.

Vis spectrum of anthraquinone radical anion

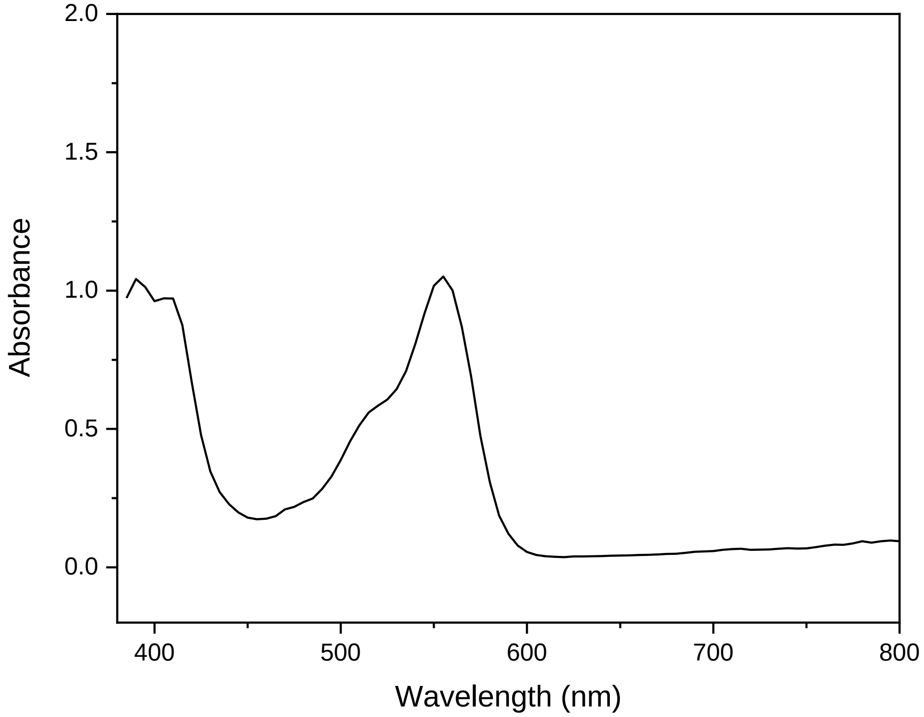

Figure S18. Vis spectrum of anthraquinone radical anion in 0.1 M Bu_4NBF_4/DMF.

Traces of anthraquinone radical anion upon addition of polymer solutions

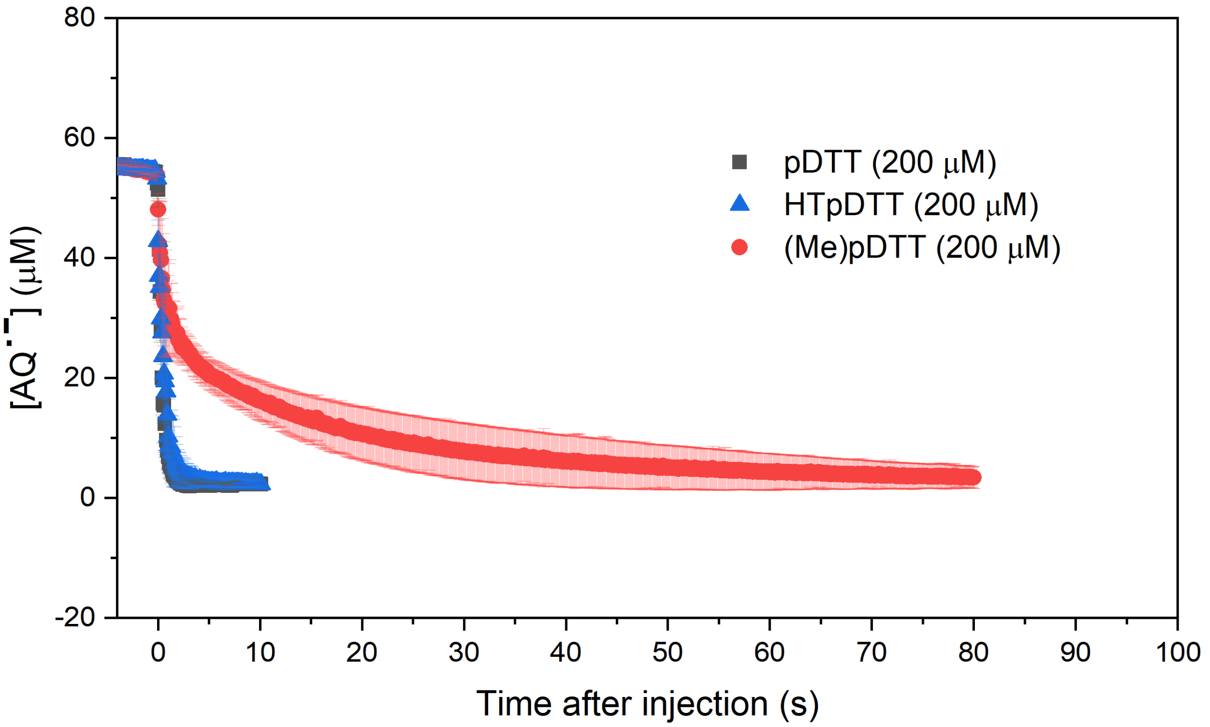

Figure S19. Traces showing change in [AQ•–] with time upon adding 100 µl polymer, resulting in 200 µM polymer concentrations (pDTT black, HTpDTT blue, (Me)pDTT red) in 0.1 M Bu_4NBF_4/DMF. Uncertainties are calculated using triplicates.

Cyclic voltammograms of polymer with and without anthraquinone

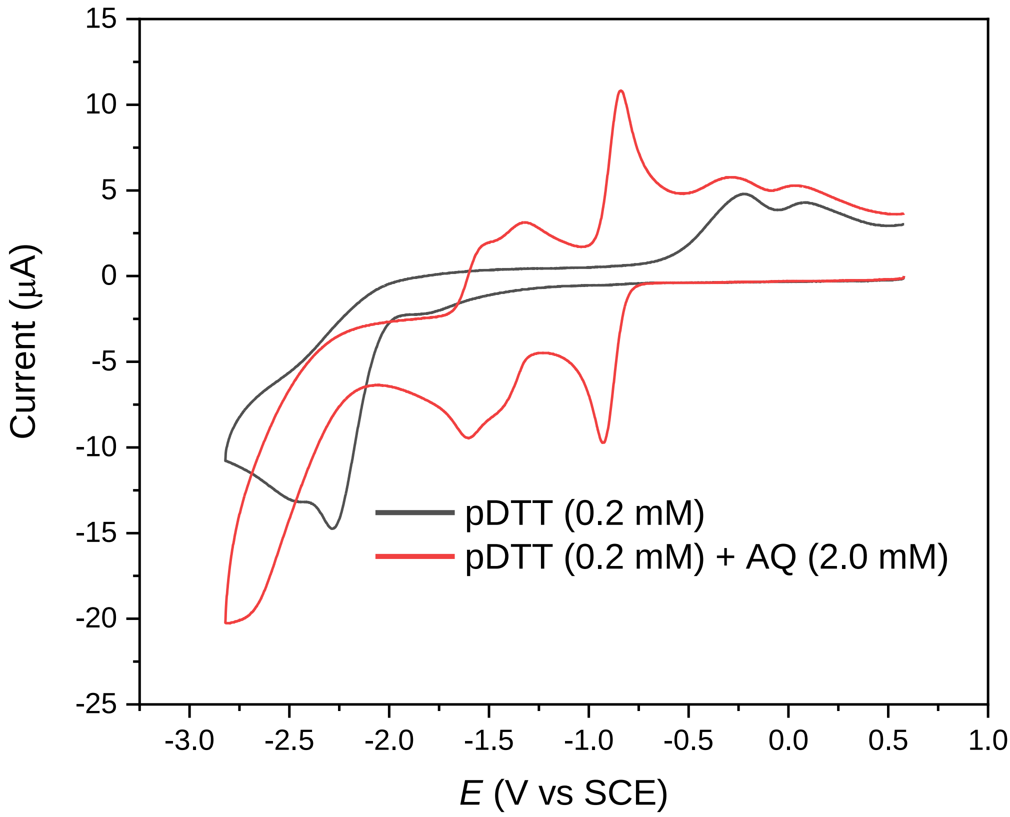

Figure S20. Cyclic voltammograms recorded at a 1 mm GC disk electrode using sweep rate of 1 V s^{-1} on pDTT (black) and pDTT/AQ (red) in 0.1 M Bu_4NBF_4/DMF. Arrows indicate scan direction from the resting potential

One of the reasons of the large increase in peak current (along with a shift in the reduction potential) could be that the smaller depolymerizing species obtained during depolymerization have higher diffusion coefficients. The emergence of two peaks during the second reduction wave, we attribute to the combined reduction of AQH^{-} and $AQ^{\bullet -}$.

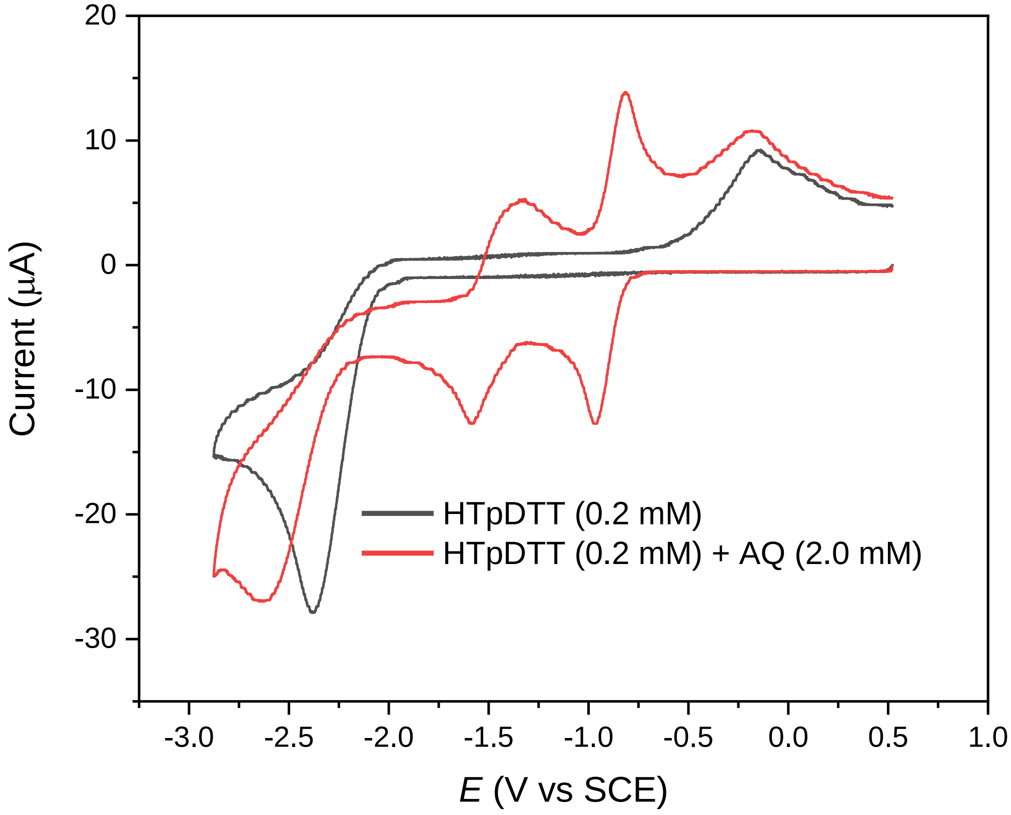

Figure S21. Cyclic voltammograms recorded at a 1 mm GC disk electrode using sweep rate of 1 V s^{-1} on HTpDTT (black) and HTpDTT/AQ (red) in 0.1 M Bu_4NBF_4/DMF. Arrows indicate scan direction from the resting potential.

Cyclic voltammetry of model compounds

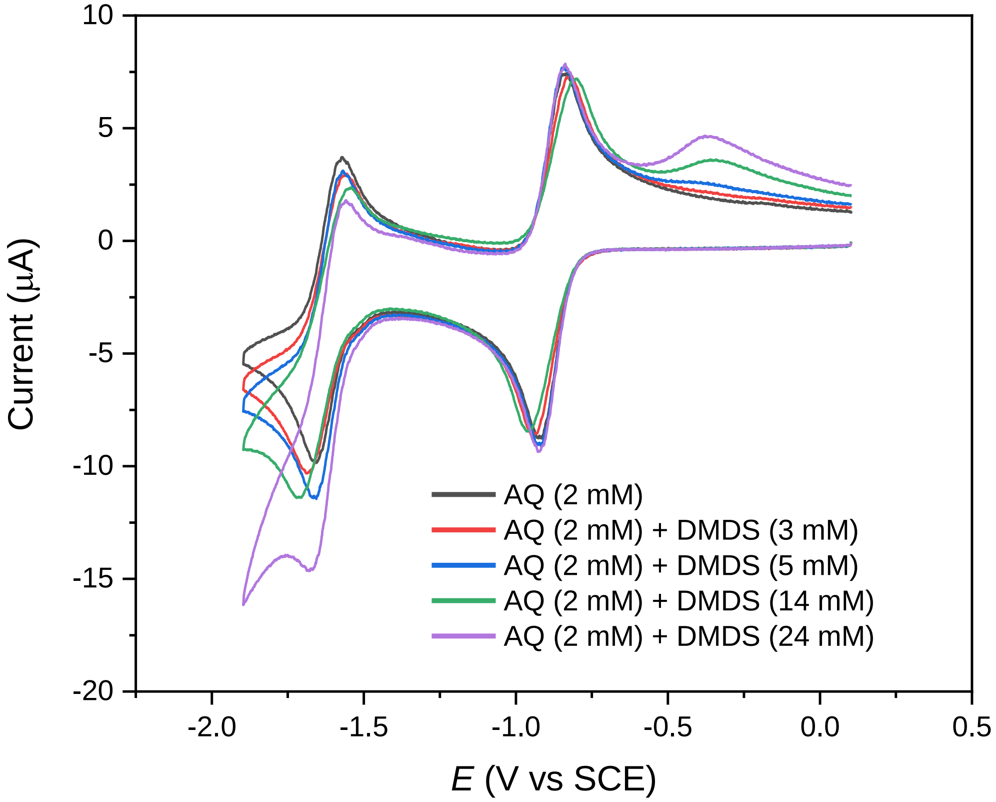

Figure S22. Cyclic voltammograms recorded at a 1 mm GC disk electrode using sweep rate of 1 V s^{-1} on AQ (black) and AQ with increasing concentrations of DMDS (red, blue, green, purple) in 0.1 M Bu_4NBF_4/DMF.

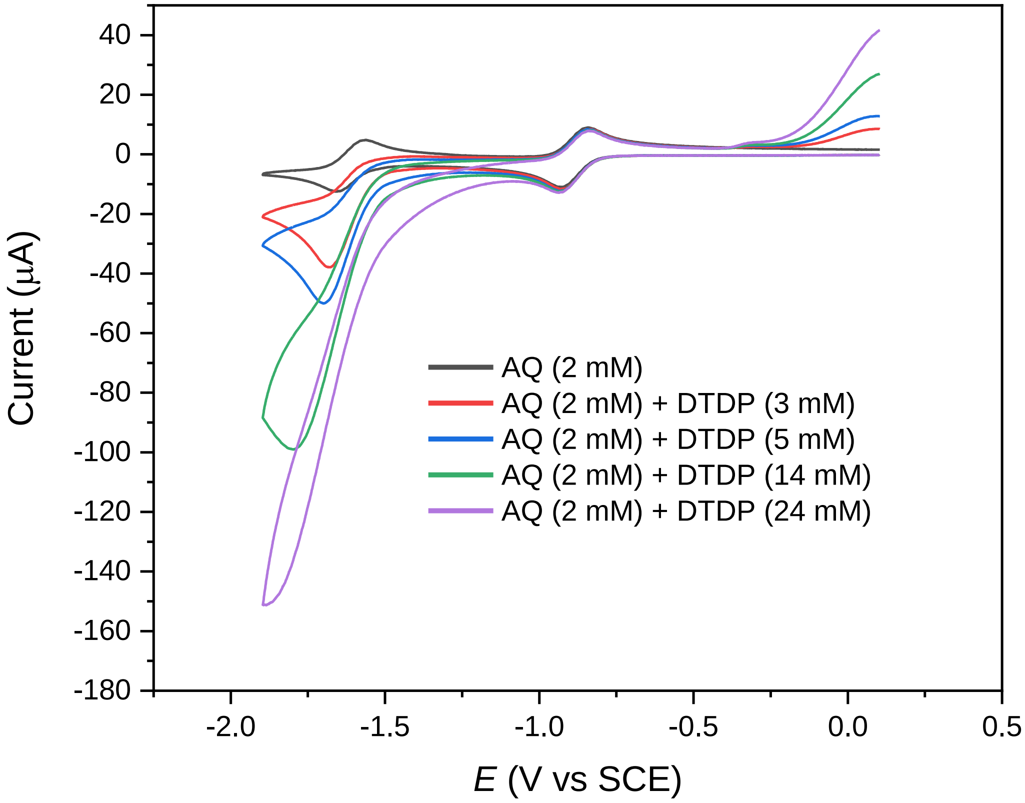

Figure S23. Cyclic voltammograms recorded at a 1 mm GC disk electrode using sweep rate of 1 V s^{-1} on AQ (black) and AQ with increasing concentrations of DTDP (red, blue, green, purple) in 0.1 M Bu_4NBF_4/DMF.

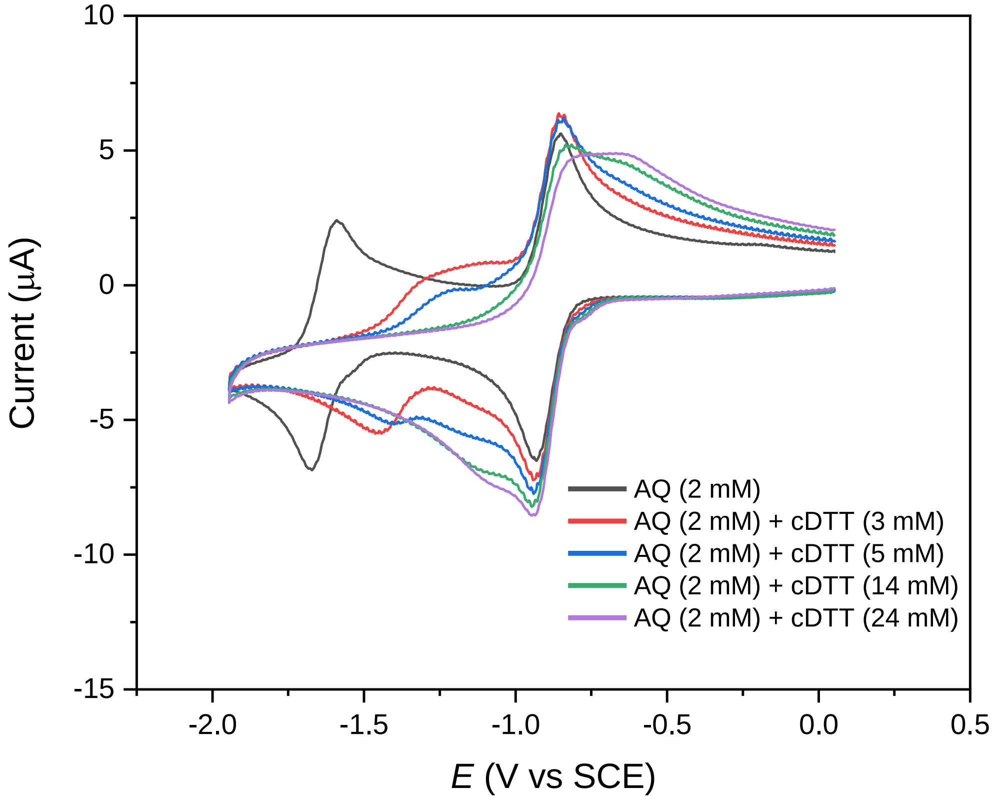

Figure S24. Cyclic voltammograms recorded at a 1 mm GC disk electrode using sweep rate of 1 V s^{-1} on AQ (black) and AQ with increasing concentrations of cDTT (red, blue, green, purple) in 0.1 M Bu_4NBF_4/DMF.

Simulated linear sweep voltammograms

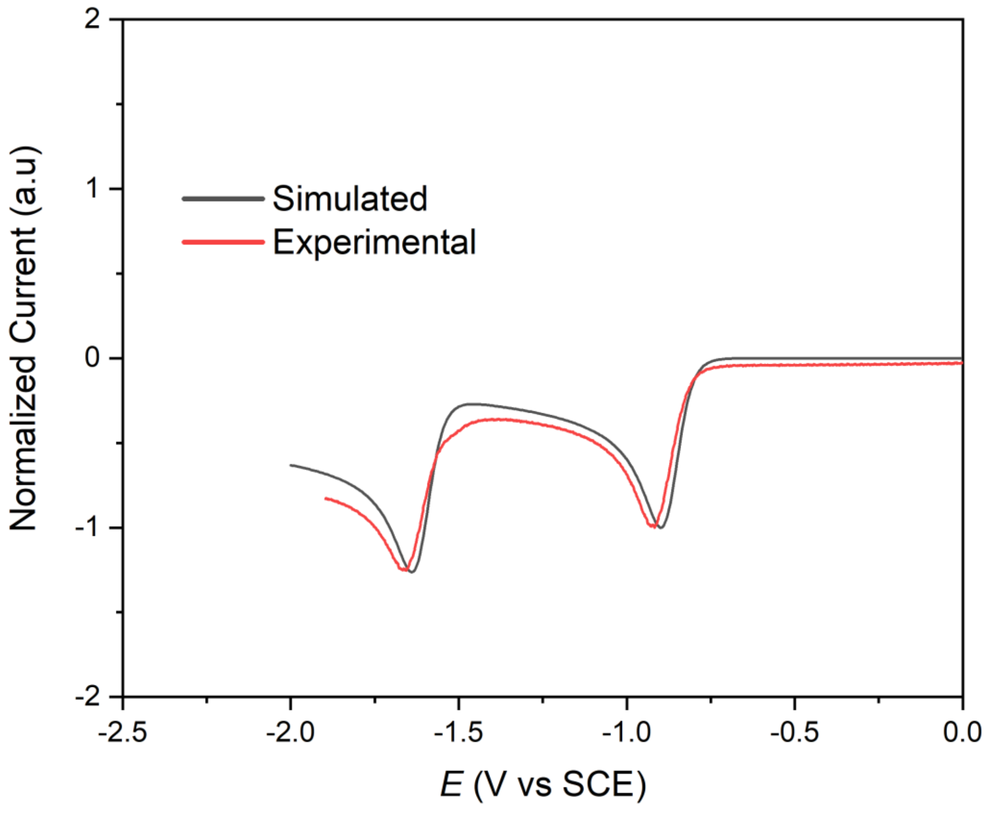

Figure S25. Linear sweep voltammograms of AQ/DMDS simulated (black) and experimental (red) with [AQ] = 2 mM, [DMDS] = 5 mM, sweep rate = 1 V s^{-1}, and k_{DMDS} = 2 × 10^2 M^{-1} s^{-1}. Current was normalized with respect to the first AQ reduction wave.

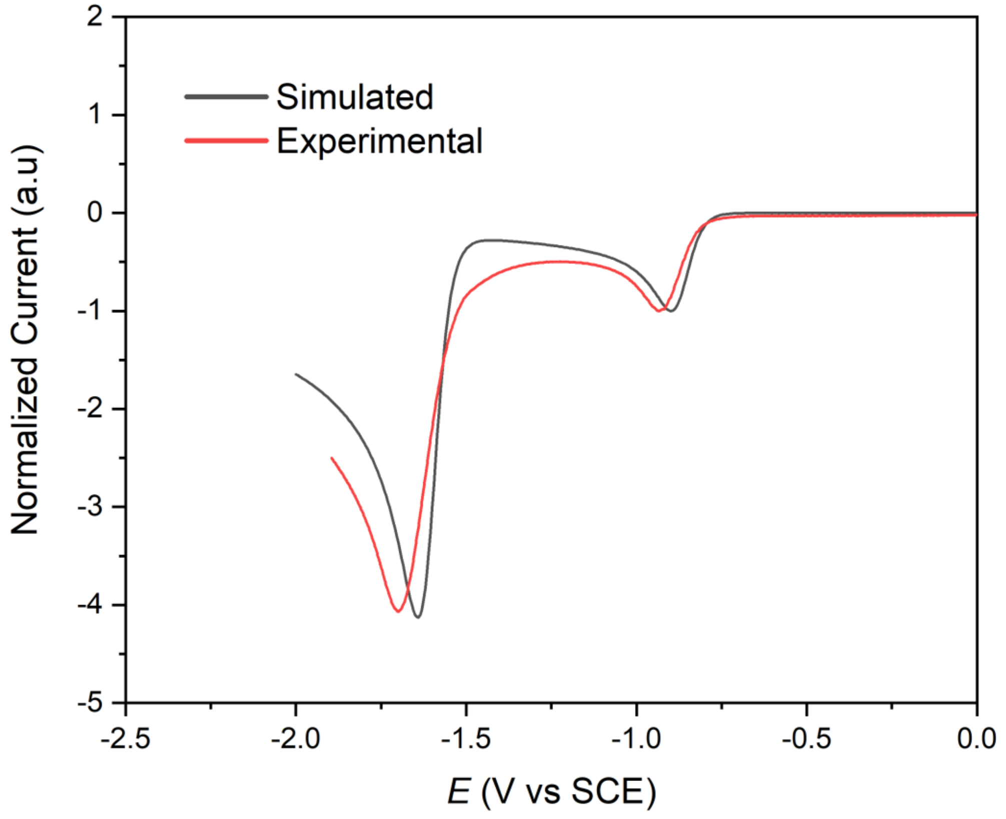

Figure S26. Linear sweep voltammograms of AQ/DTDP simulated (black) and experimental (red) with [AQ] = 2 mM, [DTDP] = 5 mM, sweep rate = 1 V s^{-1}, and k_{DTDP} = 10^5 M^{-1} s^{-1}. Current was normalized with respect to the first AQ reduction wave.

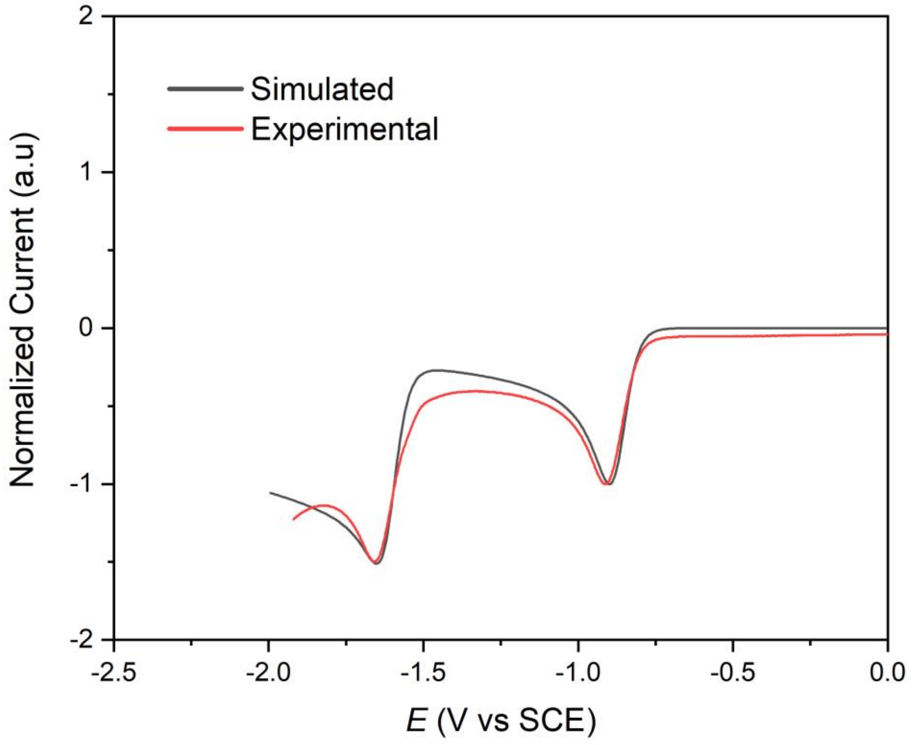

Figure S27. Linear sweep voltammograms of AQ/(Me)pDTT simulated (black) and experimental (red) with [AQ] = 2 mM, [(Me)pDTT] = 3.2 mM, sweep rate = 1 V s^{-1}, and $k_{(Me)pDTT} = 3 \times 10^3$ M^{-1} s^{-1}. Current was normalized with respect to the peak current of the first AQ reduction wave.

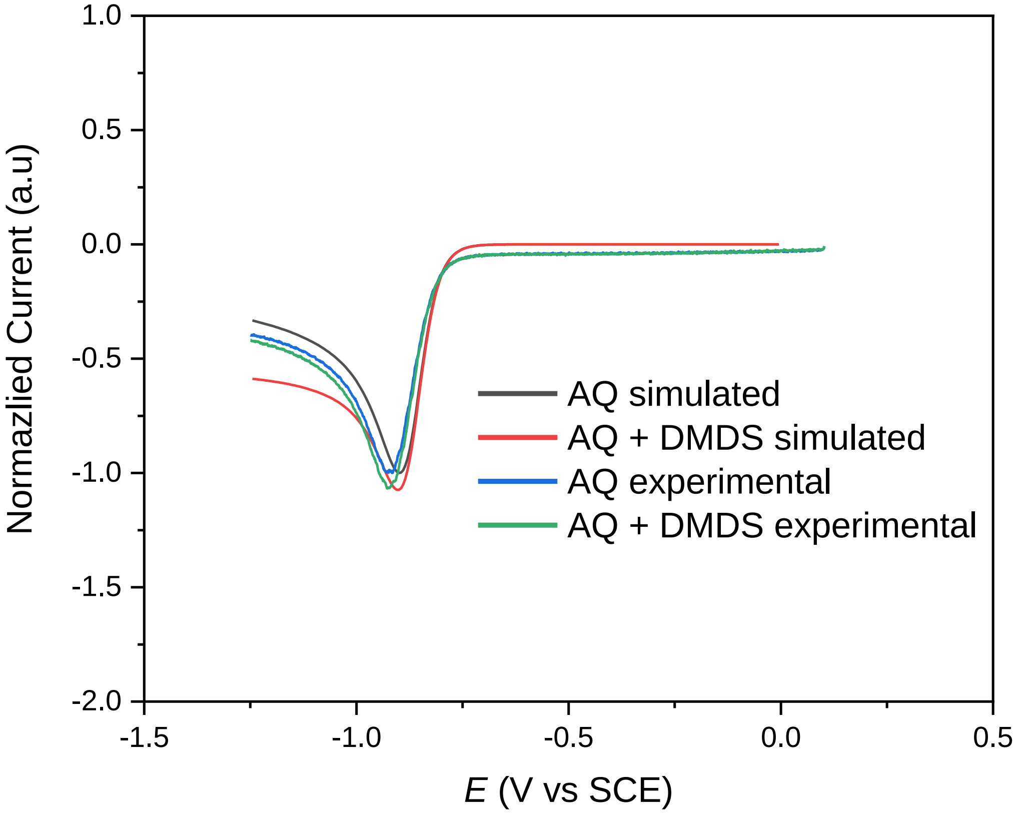

Figure S28. Linear sweep voltammograms of AQ simulated (black), AQ/DMDS simulated (red), AQ experimental (blue), and AQ/DTDP experimental (green) with [AQ] = 2 mM, [DMDS] = 24 mM, sweep rate = 1 V s^{-1}, and k_{DMDS} < 10^2 M^{-1} s^{-1}. Current was normalized with respect to the peak current of the first AQ reduction wave (without DMDS).

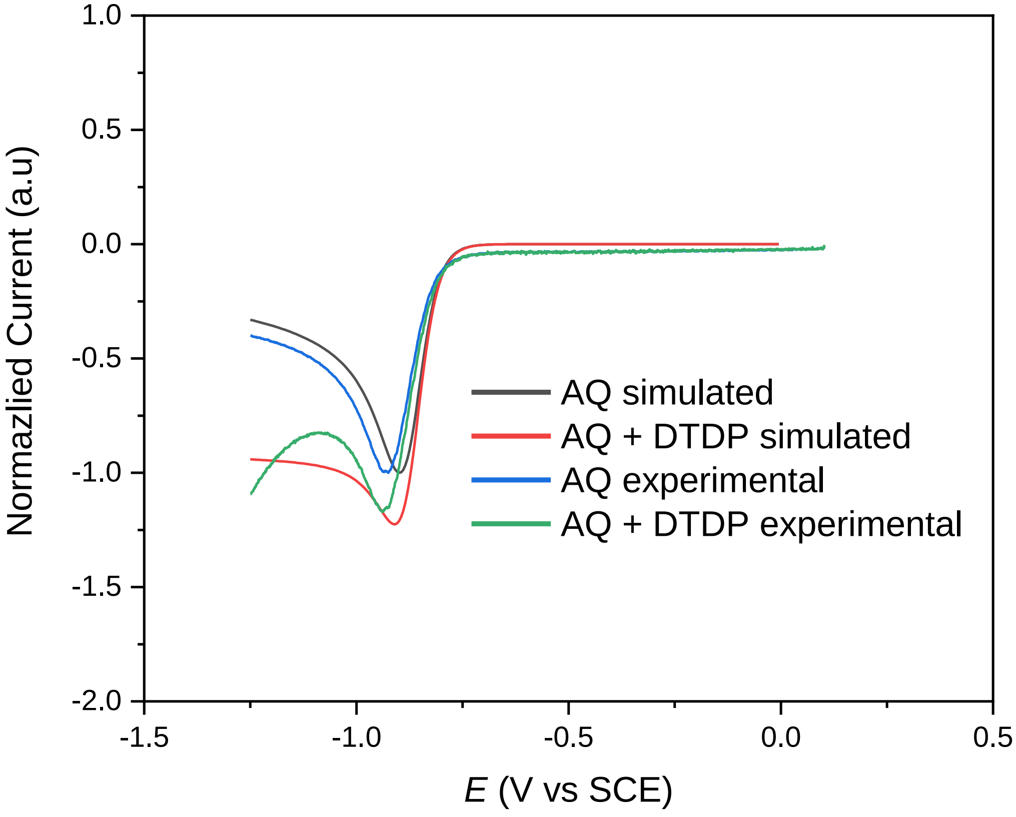

Figure S29. Linear sweep voltammograms of AQ simulated (black), AQ/DTDP simulated (red), AQ experimental (blue), and AQ/DTDP experimental (green) with [AQ] = 2 mM, [DTDP] = 24 mM, sweep rate = 1 V s^{-1}, and k_{DTDP} < 3 × 10^2 M^{-1} s^{-1}. Current was normalized with respect to the peak current of the first AQ reduction wave (without DTDP).

References

29. Hansen-Felby, M.; Henriksen, M. L.; Pedersen, S. U.; Daasbjerg, K., Postfunctionalization of Self-Immolative Poly(dithiothreitol) Using Steglich Esterification. *Macromolecules* **2022,** 55, (13), 5788–5794, 10.1021/acs.macromol.2c00551

31. Agergaard, A. H.; Sommerfeldt, A.; Pedersen, S. U.; Birkedal, H.; Daasbjerg, K., Dual-Responsive Material Based on Catechol-Modified Self-Immolative Poly(Disulfide) Backbones. *Angew. Chem. Int. Ed.* **2021,** 60, (39), 21543–21549, 10.1002/anie.202108698
